# Linking diverse salinity responses of 14 almond rootstocks with physiological, biochemical, and genetic determinants

**DOI:** 10.1038/s41598-020-78036-4

**Published:** 2020-12-03

**Authors:** Devinder Sandhu, Amita Kaundal, Biswa R. Acharya, Thomas Forest, Manju V. Pudussery, Xuan Liu, Jorge F. S. Ferreira, Donald L. Suarez

**Affiliations:** 1 USDA-ARS, U.S. Salinity Lab, 450 W Big Springs Road, Riverside, CA 92507 USA; 2 Department of Plants, Soils, and Climate, College of Agriculture and Applied Sciences (CAAS), Utah State University (USU), Logan, UT 85332 USA; 3 College of Natural and Agricultural Sciences, University of California Riverside, 900 University Avenue, Riverside, CA 92521 USA

**Keywords:** Genetics, Plant sciences

## Abstract

Fourteen commercial almond rootstocks were tested under five types of irrigation waters to understand the genetic, physiological, and biochemical bases of salt-tolerance mechanisms. Treatments included control (T1) and four saline water treatments dominant in sodium-sulfate (T2), sodium-chloride (T3), sodium-chloride/sulfate (T4), and calcium/magnesium-chloride/sulfate (T5). T3 caused the highest reduction in survival rate and trunk diameter, followed by T4 and T2, indicating that Na and, to a lesser extent, Cl were the most toxic ions to almond rootstocks. Peach hybrid (Empyrean 1) and peach-almond hybrids (Cornerstone, Bright’s Hybrid 5, and BB 106) were the most tolerant to salinity. Rootstock’s performance under salinity correlated highly with its leaf Na and Cl concentrations, indicating that Na^+^ and Cl^-^ exclusion is crucial for salinity tolerance in *Prunus*. Photosynthetic rate correlated with trunk diameter and proline leaf ratio (T3/T1) significantly correlated with the exclusion of Na^+^ and Cl^-^, which directly affected the survival rate. Expression analyses of 23 genes involved in salinity stress revealed that the expression differences among genotypes were closely associated with their performance under salinity. Our genetic, molecular, and biochemical analyses allowed us to characterize rootstocks based on component traits of the salt-tolerance mechanisms, which may facilitate the development of highly salt-tolerant rootstocks.

## Introduction

Almond [*Prunus dulcis* Miller (D.A. Webb)] is a valuable nut crop in the world. The United States is the top producer with total production exceeding 1.1 million metric tons in 2018–19 and representing more than 80% of the global share^1^. California farmers produce more than 99% of the almonds in the U.S. The total export value of the California almonds was $4.5 billion in 2017–18^2^. Although almond crop has a bigger water footprint as compared to some other crops grown in the state, it also ranks first in terms of direct economic value and is one of the most nutritionally rich food crops in seven nutrient categories^3^. Increased demand for almonds greatly expanded the almond growing area, which made farmers extend the crop cultivation to marginal soils or regions with poor irrigation water quality.

Water is the most restraining component of agriculture in the twenty-first century. Due to recurring droughts, the demand for surface water increases, which leads to increased groundwater pumping^4^. One of the biggest challenges California almond growers currently face is the irrigation water quality. Due to the increase in droughts in California, water availability is expected to decrease given groundwater aquifer subsidence as well as decreased snowpack in the Sierra Nevada mountain range, a vital storage source for California’s water system^3^. This reduced availability of good-quality water, and the increased demand for water from different sectors, is making the use of alternative or degraded waters unavoidable. Although almonds use about 12 L of water per kernel, about 43% of the water used to grow almonds in California comes from poor-quality “greywater”^3^. It is expected that to sustain the growth of California’s almond industry, the proportion of greywater must increase in the future. Different water sources that can be tapped into include treated urban effluents, runoff from greenhouse operations, and more saline groundwaters^4^. The most important consideration for the use of greywater is its salt concentration.

Salinity is one of the most important abiotic stresses critical for agricultural productivity and global food supply^5^. About 831 million hectares of the global agricultural land is affected by salinity, and predictions suggest that about half of the arable land will be impacted by 2050^5,6^. Additionally, increasing salt concentrations in irrigation water aggravate the salinity problem. The severity of the effects of salinity is regulated by several different factors such as texture, ion composition, moisture content of the soil and growth stage, species, and variety of the plant^5^. Hence, salinity is an extraordinarily complex problem.

Salt tolerance is the ability of a plant to tolerate high concentrations of salt that can be quantified in terms of growth, development, and yield. The most common way of determining the salinity tolerance of a species is by using a threshold slope model^7^. This model describes the threshold salinity of a species until which there is no considerable loss in growth and development, and the slope describes the rate at which performance declines with an increase in salinity^7^. Plants are affected by salinity in two different ways; through osmotic stress and/or ion toxicity^8,9^. In response to salinity, plants employ different strategies and mechanisms to absorb a low amount of salt, exclude excess salt, partition salt into subcellular compartments, restrict the movement of salt from roots to shoots, accumulate organic solutes in the tissue, and provide tissue tolerance to high salt concentrations^8,10,11^. Almonds are considered sensitive or moderately sensitive to salt; even a low level of salinity can lead to significant reductions in crop yield and quality^12,13^. Improving salt tolerance in almonds will not only improve yield but will also provide incentives to make augmented use of alternative/degraded waters, which may open new lands for almond cultivation. Understanding and characterizing genetic mechanisms regulating salt tolerance is crucial in developing new genetic material tolerant to salt.

In almonds, rootstock plays a key role in the success of a variety. Different types of rootstocks used in almonds include peach-based rootstocks, peach hybrids, almond-based rootstocks, plum-based rootstocks, peach/almond hybrids, plum/peach hybrids, and complex hybrids^14,15^. As the salinity tolerance of a plant depends on the ability of its roots to exclude or retain ions, in an almond variety, the salinity tolerance will largely depend on the nature of its rootstock. There is limited research conducted to evaluate a large number of rootstocks under variable salt concentrations. Based on preliminary studies in *Prunus*, significant genetic variation has been reported in different rootstocks^16–18^. However, the understanding of the relationship between this variation and its genetic determinants is still lacking. In the last few years, a range of genomic resources have been developed in the genus *Prunus*. The peach (*Prunus persica, Rosaceae*) genome has been sequenced, and expression patterns of most genes are known^19^. Genomics and bioinformatics tools have been utilized to predict putative genes and their functions^20^. However, the functional characterization of key players involved in the salt tolerance mechanism is still lacking.

The main objectives of this investigation were to evaluate almond rootstocks to determine their tolerance response to a range of saline water concentrations and compositions and to understand the morphological, physiological, biochemical, and genetic bases of salt tolerance in *Prunus*.

## Results

### Effect of salinity on survival rate

**Figure 1:**
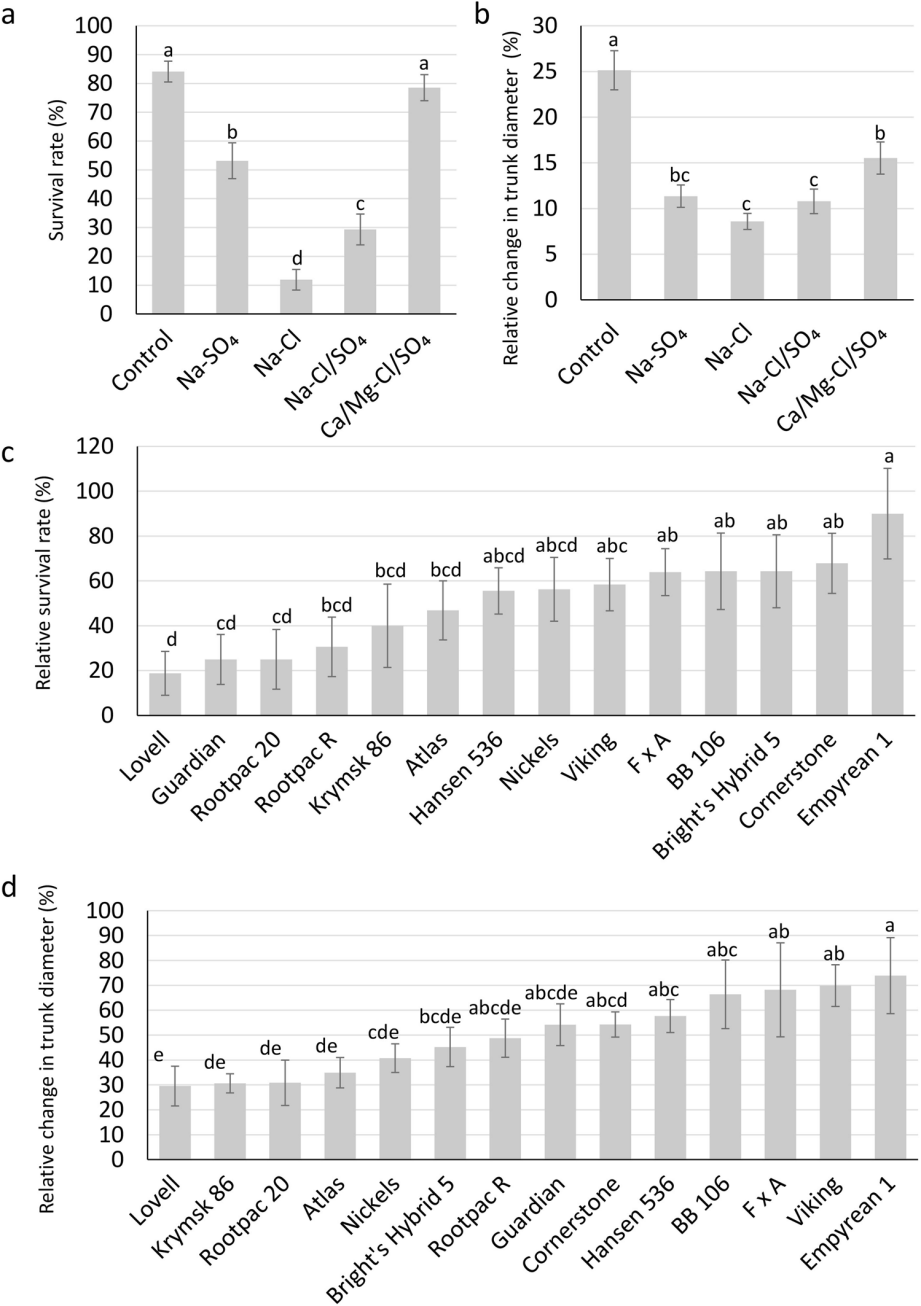
Performance of almond rootstocks in different mixed salt ion combinations of irrigation waters. (**a**) Average survival rates of all rootstocks in 5 different treatments of irrigation waters. (**b**) Relative change in trunk diameter of all rootstocks in 5 different treatments of irrigation waters. (**c**) Relative survival rates of 14 almond rootstocks in all salinity treatments (pooled data for T2–T5) with respect to control (T1). (**d**) Relative change in trunk diameter of 14 almond rootstocks in all salinity treatments (pooled data for T2–T5) with respect to control (T1). Error bars represent standard error. Means followed by the same letters are not significantly different, according to LSD (0.05).

We have analyzed fourteen (non-grafted) rootstocks irrigated with waters of mixed salt compositions to determine their ability to tolerate salinity. Treatments included control (T1), Na-SO_4_ dominant (T2), Na-Cl dominant (T3), Na-Cl/SO_4_ dominant (T4), and Ca/Mg-Cl/SO_4_ dominant irrigation waters (T5) (Table 1). Ten months after the initiation of salt treatments, rootstocks were analyzed for their survival rates and leaf toxicity symptoms. The T3 treatment was the harshest treatment for the plants with the lowest average survival rate (11.90%) followed by T4 (29.37%), T2 (53.17%), T5 (78.57%), and T1 (84.13%) (Fig. 1a). Empyrean 1 displayed a high relative survival rate (90.0%) for four salinity treatments, followed by Cornerstone (67.9%), Bright’s Hybrid 5 (64.3%), BB106 (64.3%), F x A (63.9%), and Viking (58.3%) (Fig. 1c and Supplementary Fig. S1). On the other hand, Lovell (18.8%), Guardian (25.0%), and Rootpac 20 (25.0%) had low average survival rates for the four salinity treatments. While many of the rootstocks had high mortality rates in Na-Cl dominant treatment, Empyrean 1 had minimal toxicity symptoms on the leaves.

**Table 1:** Composition of the irrigation water.

Treatment name	Treatment description
Control (T1)	Non-saline control {Na^+^ 1.65 meq L^−1^, K^+^ 6.5 meq L^−1^, PO4^3−^ 1.5 meq L^−1^, Mg^2+^ 1.3 meq L^−1^, SO_4_^2−^ 1.5 meq L^−1^, Cl^−^ 1.5 meq L^−1^, NO3^−^ 5 meq L^−1^ and micronutrients}
Na-SO_4_ dominant (T2)	Mixed cations (Ca^2+^ = 1.25 Mg^2+^ = 0.25 Na^+^) with predominantly sulfate (Cl^−^ = 0.2 SO_4_^2−^) {Na^+^ 18 meq L^−1^, Ca^2+^ 4.5 meq L^−1^, K^+^ 6.5 meq L^−1^, PO4^3−^ 1.5 meq L^−1^, Mg^2+^ 3.6 meq L^−1^, SO_4_^2−^ 22 meq L^−1^, Cl^−^ 4.4 meq L^−1^, NO3^−^ 5 meq L^−1^ and micronutrients}
Na-Cl dominant (T3)	Mixed cations (Ca^2+^ = 1.25 Mg^2+^ = 0.25 Na^+^) with predominantly chloride (SO_4_^2−^ = 0.2 Cl^−^) {Na^+^ 15.5 meq L^−1^, Ca^2+^ 3.8 meq L^−1^, K^+^ 6.5 meq L^−1^, PO4^3−^ 1.5 meq L^−1^, Mg^2+^ 3.1 meq L^−1^, SO_4_^2−^ 3.8 meq L^−1^, Cl^−^ 19 meq L^−1^, NO3^−^ 5 meq L^−1^ and micronutrients}
Na-Cl/SO_4_ dominant (T4)	Mixed anions SO_4_-Cl (SO_4_^2−^ = Cl^−^), predominantly Sodium (Ca^2+^ = 1.25 Mg^2+^ = 0.25 Na^+^) {Na^+^ 17 meq L^−1^, Ca^2+^ 4.25 meq L^−1^, K^+^ 6.5 meq L^−1^, PO4^3−^ 1.5 meq L^−1^, Mg^2+^ 3.4 meq L^−1^, SO_4_^2−^ 12.32 meq L^−1^, Cl^−^ 12.32 meq L^−1^, NO3^−^ 5 meq L^−1^ and micronutrients}
Ca/Mg-Cl/SO_4_ dominant (T5)	Mixed anions SO_4_^2−^-Cl^−^ (SO_4_^2−^ = Cl^−^), predominantly Ca^2+^ and Mg^2+^ (Ca^2+^ = 1.25 Mg^2+^ = 5 Na^+^) {Na^+^ 2.75 meq L^−1^, Ca^2+^ 13.5 meq L^−1^, K^+^ 6.5 meq L^−1^, PO4^3−^ 1.5 meq L^−1^, Mg^2+^ 10.8 meq L^−1^, SO_4_^2−^ 13.5 meq L^−1^, Cl^−^ 13.5 meq L^−1^, NO3^−^ 5 meq L^−1^ and micronutrients}

### Effect of salinity on trunk diameter

Trunk diameter is also an important parameter that is used to determine plant growth over time. We used a change in trunk diameter as a parameter of growth under salinity. Before initiating salinity treatments, trunk diameter was measured for all the plants in July 2017. Trunk diameter was again measured in May 2018, and the relative percent change in trunk diameter was calculated relative to the start of the experiment (Fig. 1b). Na-Cl dominant treatment showed the lowest change in trunk diameter (8.59%) followed by Na-Cl/SO_4_ (10.79%), Na-SO_4_ (11.35%), Ca/Mg-Cl/SO_4_ (15.54%) and control (25.13%) (Fig. 1b). The smaller increase in diameter in all three Na-dominant treatments (Na-Cl dominant, Na-Cl/SO_4_ dominant, and Na-SO_4_ dominant) indicated the importance of Na^+^ toxicity in almonds. A smaller increase in trunk diameter in Na-Cl dominant treatment versus Na-SO_4_ dominant treatment suggested the importance of both Na^+^ and Cl^-^ toxicities during salt stress (Fig. 1b).

Of the 14 tested rootstocks, Empyrean 1 had the highest relative change in trunk diameter (73.89%) (Fig. 1d and Supplementary Fig. S1). Other rootstocks that performed well in salinity treatments with respect to trunk diameter include Viking, F x A, BB 106, Hansen 536, and Cornerstone (Fig. 1d and Supplementary Fig. S1). The rootstocks that performed the worst under all salinity treatments were Lovell, Krymsk 86, and Rootpac 20.

### Effect of salinity on leaf ion composition

**Figure 2:**
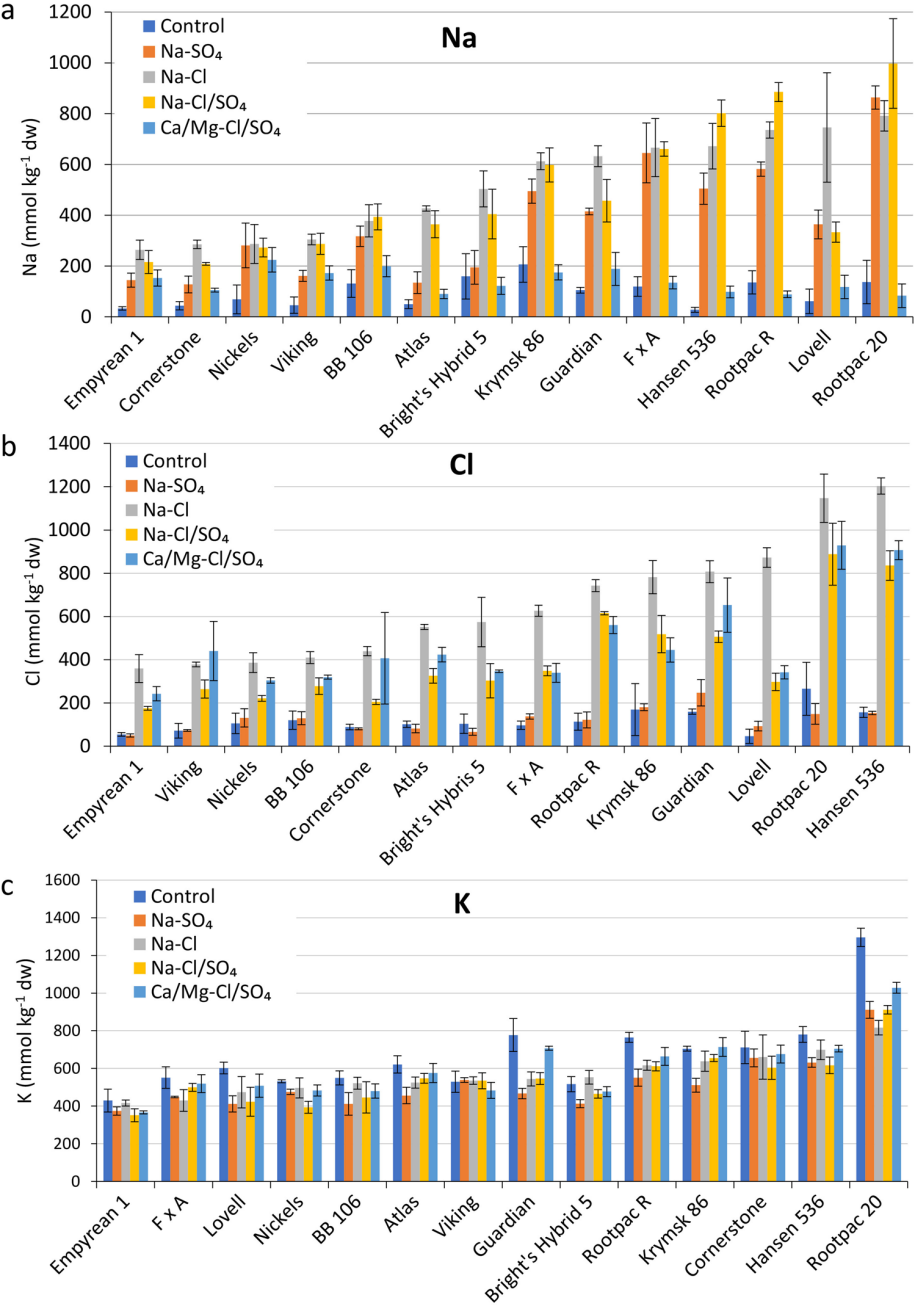
Leaf ion concentrations of 14 almond rootstocks subjected to 5 irrigation water treatments. (**a**) Leaf Na concentrations. (**b**) Leaf Cl concentrations. (**c**) Leaf K concentrations. Error bars represent standard errors of three biological replicates. Rootstocks are arranged on the *x*-axis in ascending order based on T3 (Na-Cl dominant treatment) for their shoot Na, Cl, and K accumulations.

Ion analysis was performed on digested leaf samples for Na, Cl, K, Ca, Mg, P, S, B, Cu, Fe, Mn, Mo, and Zn (Supplementary Table S1). Of the five salinity treatments, Na-Cl dominant treatment (T3) led to a maximum accumulation of Na in leaves (Fig. 2a). The average leaf Na concentrations ranged from 27.8 to 206.3 mmol kg^−1^ dry weight (dw) under control (T1) and 263.7 to 791.4 mmol kg^−1^ dw under Na-Cl dominant treatment (T3). Under Na-Cl dominant treatment, Empyrean 1 stored the least amount of Na in leaves (Fig. 2a). Other rootstocks that stored a low Na include Cornerstone, Nickels, Viking, BB 106, Atlas, and Bright’s Hybrid 5 (Fig. 2a). Interestingly, Hansen 536, which is one of the low Na accumulators under control (T1), had a more than 23-fold average increase in three Na dominant treatments (T2, T3, and T4) (Fig. 2a).

Of the five different treatments, Na-Cl dominant treatment led to a maximum accumulation of Cl in leaves. The average leaf Cl concentrations ranged from 46.2 to 266.0 mmol kg^−1^ dw under control (T1) and 359.2 to 1203.2 mmol kg^−1^ dw under Na-Cl dominant treatment (T3) (Fig. 2b). Under the control treatment (T1), Lovell stored the least amount of Cl in leaves, followed by Empyrean 1, Viking, and Cornerstone. Interestingly, Lovell was among the top three Cl accumulators in Na-Cl dominant treatment (T3) (Supplementary Table S1). In four salinity treatments (T3-T5), on an average, Empyrean 1 stored the least amount of Cl, followed by Nickels, BB 106, Cornerstone, and Viking (Fig. 2b and Supplementary Table S1). Rootpac 20, Hansen 536, Guardian, and Rootpac R were high Cl accumulators under salinity treatments.

In most rootstocks, the total K content in the leaves was reduced in all four salinity treatments compared to the control (Fig. 2c). The maximum reduction was in Na-SO_4_ dominant treatment (T2). Viking showed the least reduction (1.2%) in four salinity treatments compared to the control, followed by Bright’s Hybrid 5 (7.8%) and Cornerstone (8.7%) (Supplementary Table S1). Empyrean 1, one of the top-performing genotypes, had a 12.2% reduction in leaf K concentration under salinity compared to the control. The rootstocks that showed maximum reduction included Rootpac 20 (29.3%), Guardian (27.2%), Lovell (24.6%), and Rootpac R (20.2%) (Fig. 2c and Supplementary Table S1). Although of all the rootstocks, Rootpac 20 stored the most amount of K in leaf tissue under T1, it exhibited the highest K reduction in all four salinity treatments (T2 – T5) compared to the other rootstocks (Supplementary Table S1).

For other ions including Ca, Mg, P, S, B, Cu, Fe, Mn, Mo, and Zn, shoot concentration varied significantly among different rootstocks; however, there was no clear correlation with the survival rate or trunk diameter (Supplementary Table S1).

### Effect of salinity on gas exchange parameters

**Figure 3:**
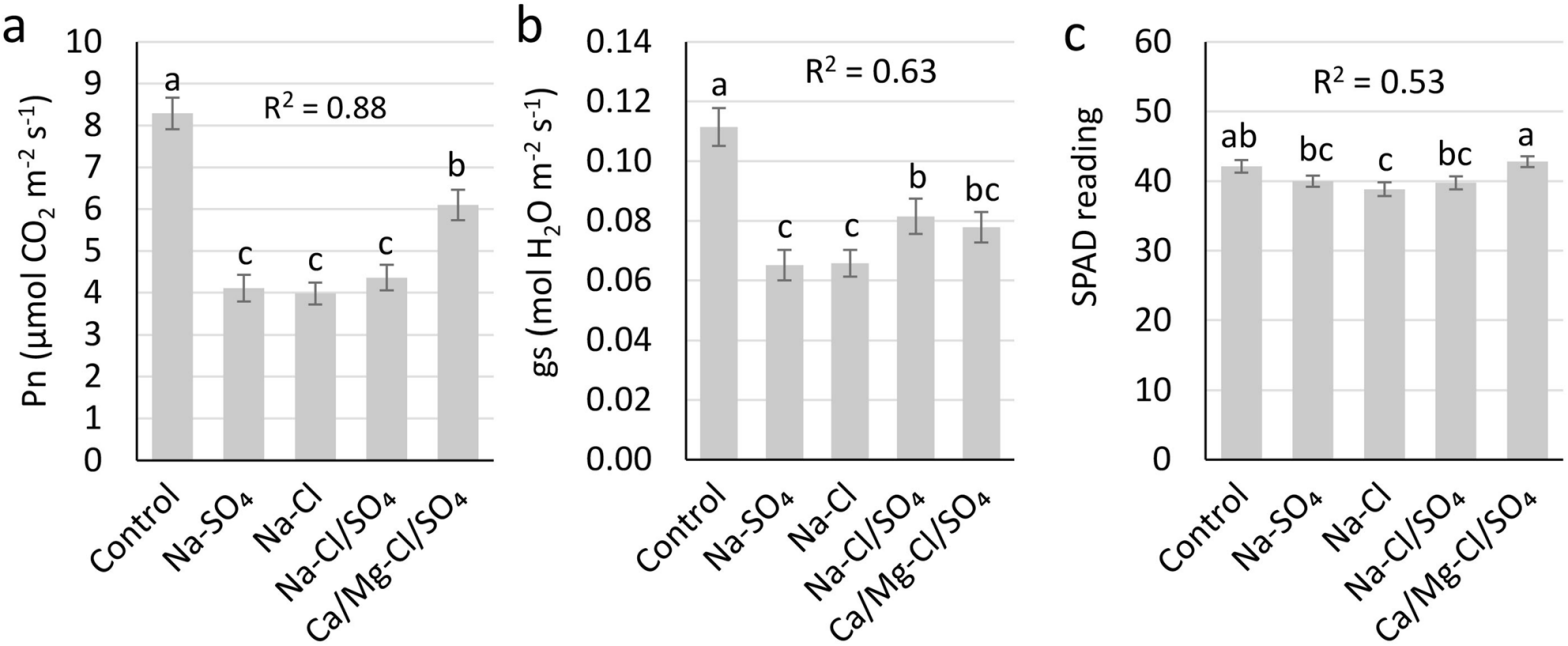
Physiological leaf measurements in almond rootstocks under different salt treatments. Data for all the rootstocks were pooled for each treatment. (**a**) Net photosynthesis (*Pn*); (**b**) stomatal conductance (*gs*); (**c**) *SPAD* reading for Chlorophyll content. The correlation of each trait with the trunk diameter is represented as R^2^. Error bars represent standard error. Means followed by the same letters are not significantly different, according to LSD (0.05).

The analyses of physiological parameters for 14 almond rootstocks treated with five treatments of irrigation waters suggested that there were significant differences among treatments and rootstocks (*P* < 0.001) for the net photosynthetic rate (*Pn*), leaf stomatal conductance (*gs*), and Soil–Plant Analysis Development (*SPAD*) chlorophyll content. T3 showed maximum reduction for *Pn*, *gs*, and *SPAD* as compared to the control (T1) (Fig. 3). On the other hand, T5 had the least reduction in *Pn* and *SPAD* as compared to the control, and T4 exhibited the least reduction for *gs*.

A comparison of different rootstocks in T3 revealed that Nickels, Empyrean 1, Hansen 536, and Bright’s Hybrid 5 had high *Pn* values as compared to the other rootstocks (Supplementary Table S2). Lovell was the worst performer for *Pn* in T3. Hansen 536 performed well in all four salinity treatments (T2–T5) (Supplementary Table S2). Nickels, Empyrean 1, and Hansen 536 were also the top three performers for *gs* in T3, whereas Lovell and Guardian were the two worst performers (Supplementary Table S2). For the *SPAD* chlorophyll content, Hansen 536, Viking, Bright’s Hybrid 5, Cornerstone, and Nickels were good performers in T3 (Supplementary Table S2). Rootpac 20 and Guardian, and Rootpac R were the worst performers. Hansen 536 and Viking were two top performers for *SPAD* readings among all four salinity treatments (Supplementary Table S2).

### Effect of salinity on biochemical responses

The biochemical responses of 14 rootstocks were evaluated under different salinity treatments. The biochemical markers chosen were proline, antioxidant capacity (ORAC), and total phenolics in leaves of rootstocks exposed to different treatments. The ORAC values ranged from 838.7 (Hansen 536) to 3069.5 µmoles TE g^−1^ dw (Lovell) and total phenolics ranged from 19.5 (Hansen 536) to 47.7 mg GAE g^−1^ dw (Krymsk 86) (Supplementary Table S3). The ratio of Na-Cl dominant treatment and control (T3/T1) for ORAC and total phenolics displayed  R^2^ value of 0.32 and 0.20 with survival rate, respectively (data not shown).

**Figure 4:**
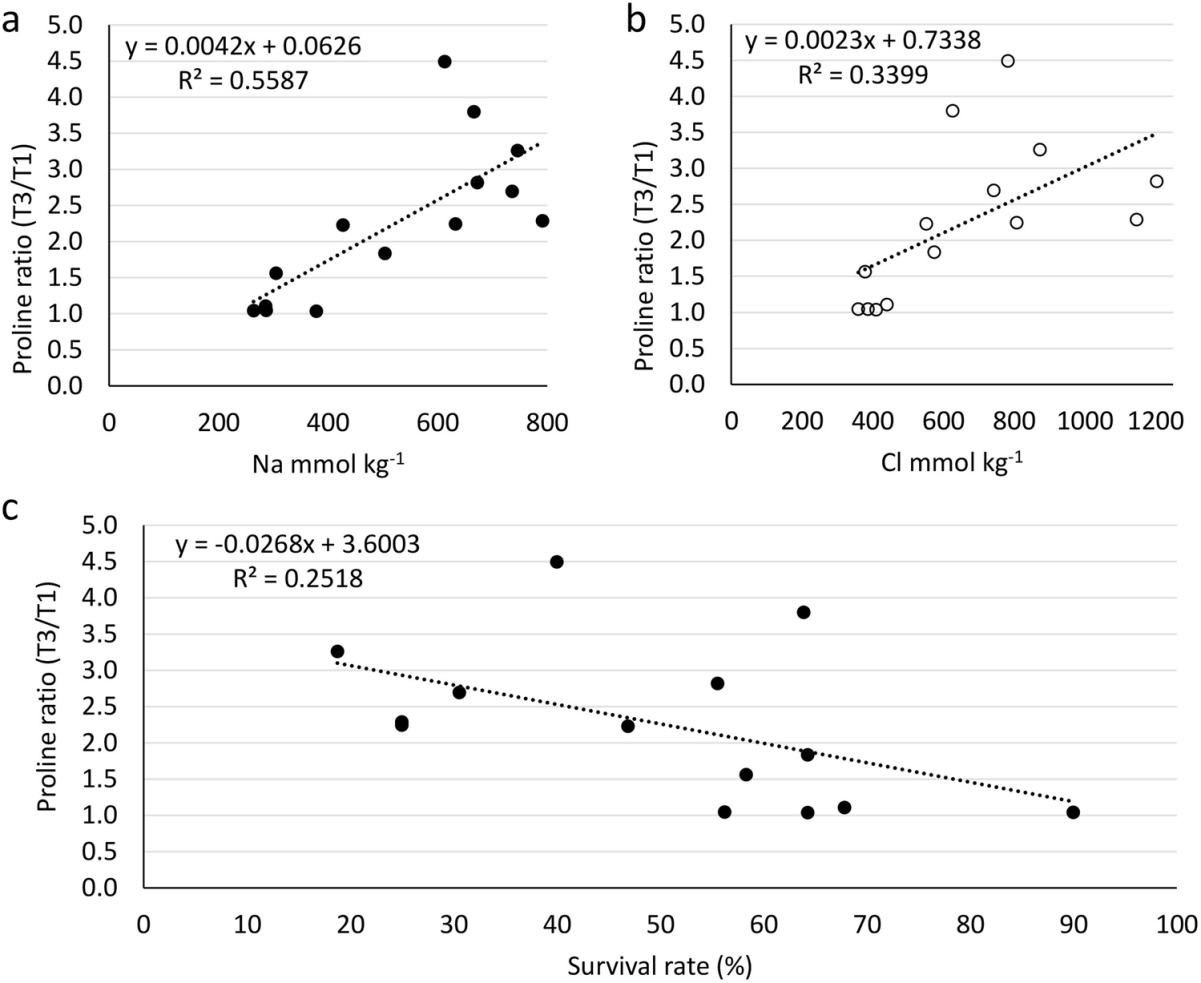
Relationship of tissue proline concentrations with leaf Na and Cl concentrations and survival rate in 14 almond rootstocks. (**a**) The relationship between a ratio of proline concentration in Na–Cl dominant treatment and control (T3/T1) with leaf Na concentration. (**b**) The relationship between a ratio of proline concentration in Na-Cl dominant treatment and control (T3/T1) with leaf Cl concentration. (**c**) The relationship between a ratio of proline concentration in Na–Cl dominant treatment and control (T3/T1) with leaf Cl concentration. Dots represent mean values of different rootstocks, n = 3.

Leaf proline concentrations at control salinity (T1) ranged from 1.0 (Krymsk 86) to 3.1 mg g^−1^ dw (BB 106), while under Na-Cl dominant irrigation water treatment (T3) proline ranged from 1.5 (Nickels) to 7.3 mg g^−1^ dw (Lovell) (Supplementary Table S4). Relative proline ratio in the leaves of the 14 rootstocks, expressed as a ratio of the Na-Cl dominant treatment and the control (T3/T1), ranged from 1.04 for Empyrean 1 and BB 106 to 4.50 for Krymsk 86 (Fig. 4 and Supplementary Table S4). The rootstocks with the low leaf proline ratio (T3/T1) such as Empyrean 1 (1.04), Cornerstone (1.11), Nickels (1.05), and BB 106 (1.04) were the top Na and Cl excluders (Supplementary Table S4). On the other hand, rootstocks with the high proline ratio, such as Krymsk 86 (4.50), F x A (3.80), and Lovell (3.26), were low Na and Cl excluders (Supplementary Table S4). Proline ratio had a significant positive correlation with leaf Na (R^2^ = 0.56) and Cl (R^2^ = 0.34) concentrations (Fig. 4a and b). An inverse correlation of R^2^ = 0.25 was found between the proline ratio and the survival rate (Fig. 4c). The four rootstocks with the best survival rates (Empyrean 1, Cornerstone, Bright’s Hybrid 5, and BB 106) had the lowest proline ratios (T3/T1) ranged from 1.04 to 1.11, while the four rootstocks with the worse survival rates (Lovell, Guardian, Rootpac 20, and Rootpac R) had proline ratios ranging from 2.3 to 3.3 mg g^−1^ dw (Supplementary Table S4).

### Effect of salinity on gene expression

**Figure 5:**
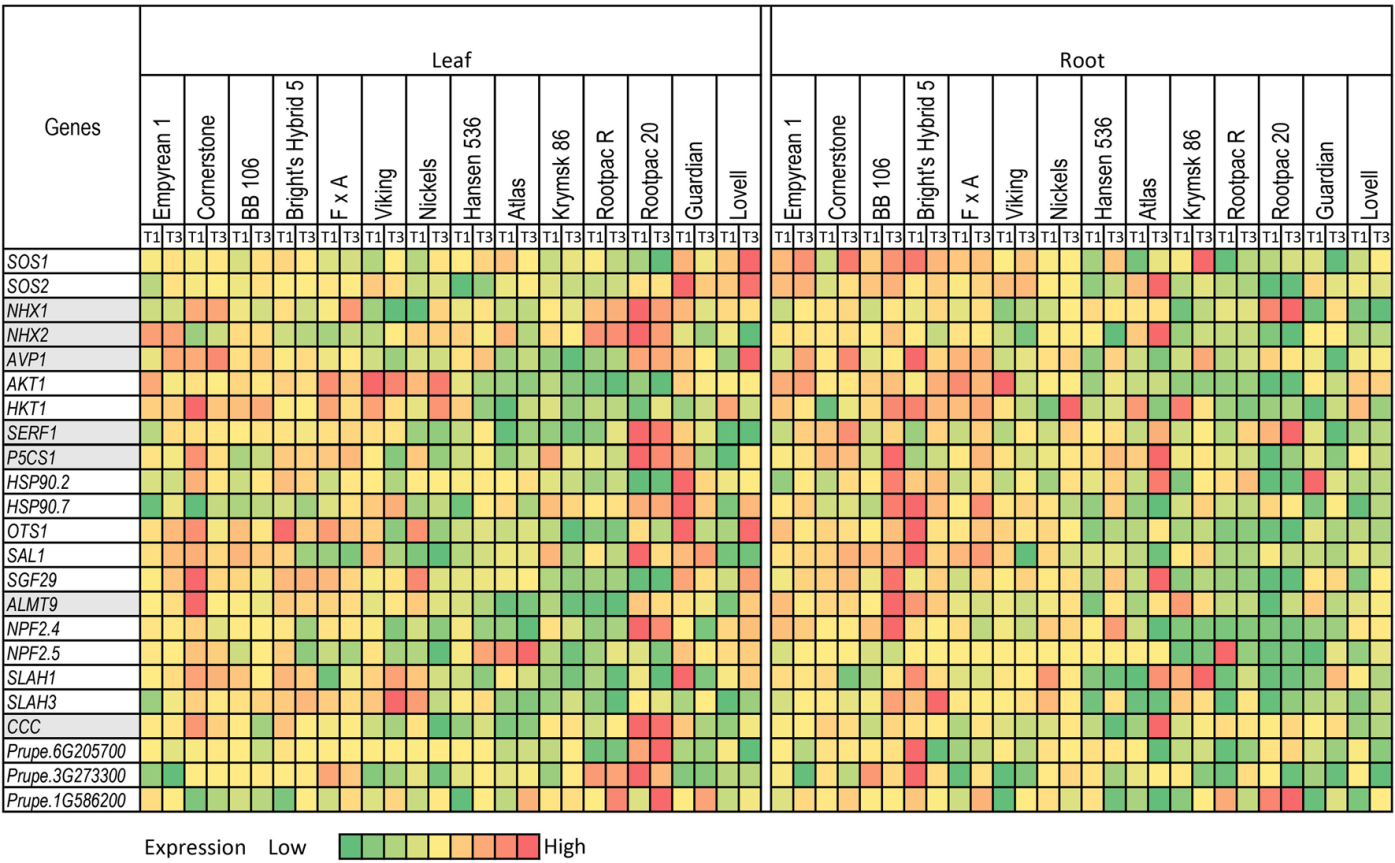
Heatmap representing relative expressions of salt-stress related genes in roots and leaves of 14 almond rootstocks under control (T1) and Na–Cl dominant treatment (T3). Expression values for each gene are color-coded to depict the fold-change in different rootstock. Genotypes are arranged on the *x*-axis in descending order of their salt tolerance.

Expression analyses were carried out for a set of 23 genes selected for their involvement in salt stress. The expression analyses on 14 different genotypes revealed that the basal expression of most genes, both under control (T1) and Na-Cl dominant salinity treatment (T3), were higher in roots of the salt-tolerant compared to the salt-sensitive genotypes (Fig. 5). For instance, in T3, the *SOS1* and *SOS2* genes expressed at higher levels in roots of Empyrean 1, Cornerstone, BB 106, and Bright’s Hybrid 5 compared to Lovell, Guardian, and Rootpac R (Fig. 5). Similarly, three genes *AKT1*, *OTS1*, and *SAL1*, had expression levels several-fold higher in the top 5 salt-tolerant genotypes than in the salt-sensitive ones (Fig. 5 and Supplementary Table S5).

Comparisons among the four most salt-tolerant genotypes revealed differences in expression levels of various genes. Empyrean 1 displayed high expression levels for most genes in roots under salinity except *NPF2.5*, *Prupe.6G205700*, and *Prupe.3G273300* (Fig. 5). Cornerstone had high expression levels for *SOS1*, *AVP1*, *SERF1*, *P5CS1*, *SAL1*, and *SGF29* in roots in T3, whereas the expression levels were low for *HSP90.7, SLAH1,* and *Prupe.3G273300*. BB 106 displayed high expression levels for most genes in roots of plants under T3, except *NPF2.5* (Fig. 5). Bright’s Hybrid 5 had low expression levels for *NHX2, P5CS1*, and *Prupe.6G205700*. Expression levels of *Prupe.3G273300* were hundreds of folds higher in BB 106 and Bright’s Hybrid 5 than in Empyrean 1 and Cornerstone in roots of plants under T3 (Fig. 5 and Supplementary Table S5). Similarly, although the salt-sensitive genotypes displayed lower expression levels for most genes, there was a significant variation among them. For instance, Lovell had a relatively high expression of *AKT1*, Guardian had a high expression of *NHX1* and *NPF2.5,* and Rootpac R had a high expression of *SERF1* and *HSP90.2* in roots in T3 (Fig. 5).

In roots, the *SERF1* and *SGF29* genes were significantly upregulated in T3 compared to T1 in the top four salt-tolerant lines (Fig. 5). *P5CS1* and *SLAH3* were induced under salinity (T3) among three of the top four salt-tolerant lines. *HKT1*, *P5CS1*, *HSP90.2*, *HSP90.7*, *SGF29*, *ALMT9*, *NPF2.4*, *SLAH1*, *SLAH3*, and *CCC* all had at least two-fold upregulation in T3 compared to T1 in BB 106, one of the salt-tolerant genotypes (Fig. 5 and Supplementary Table S5). *HKT1* was highly induced (6.7x) in T3 compared to T1 in Cornerstone. Lovell, one of the most salt-sensitive genotype, showed significant downregulation for *P5CS1* and *SLAH1* in roots in T3 compared to T1 (Fig. 5). Krymsk 86 and Lovell exhibited more than 2.5-fold downregulation for *HKT1* in roots in T3 compared to T1. Guardian had more than two-fold reduction in expression in roots in T3 compared to T1 for *SOS1*, *AVP1*, *SERF1*, *HSP90.2*, and *ALMT9* (Fig. 5 and Supplementary Table S5). Rootpac R roots displayed more than 200,000-fold downregulation for NFP2.5 and more than a three-fold reduction for *Prupe.1G586200* in T3 compared to T1 (Supplementary Table S5).

Although the overall trend of high expression in salt-tolerant genotypes was maintained for most genes in leaves, it was not as clear as in roots (Fig. 5). Under salinity (T3), Empyrean 1 had high expressions for *NHX2*, *AVP1*, *SERF1*, and *SAL1*; Cornerstone showed high expressions for *NHX1*, *AVP1*, and *SAL1*; and BB106 had high expressions for *HKT1*, *SERF1*, and *SAL1* in leaves (Fig. 5). On the other hand, salt-sensitive genotypes, Krymsk 86 and Rootpac R exhibited low expressions for *AVP1*, *AKT1*, *HKT1*, *HSP90.2*, *OTS1*, *SAL1*, *SGF29*, *ALMT9*, *SLAH1*, and *SLAH3* under salinity. Guardian and Lovell exhibited low expressions for *NHX2*, *HKT1*, *Prupe.6G20700*, and *Prupe3G273300* (Fig. 5). Although, in general, the salt-sensitive genotypes had relatively low expression for several genes under salinity in leaves, some genes were highly expressed in these genotypes. For instance, Rootpac R had relatively high expressions of *NHX1*, *NHX2*, *Prupe3G273300*, and *Prupe.1G586200* and Guardian displayed high expressions for *HSP90.7*, *SAL1*, and *Prupe.1G586200*. Interestingly, Lovell exhibited high expressions for several genes in leaves, including *SOS1*, *SOS2*, *AVP1*, *HSP90.7*, *OTS1*, *SGF29*, and *NPF2.4* (Fig. 5).

In leaves, a few genes were significantly upregulated in salt-tolerant varieties and downregulated in salt-sensitive varieties under salinity (Fig. 5 and Supplementary Table S5). For instance, *AVP1* was significantly upregulated in Empyrean 1, Cornerstone, BB 106, and Bright’s Hybrid 5. *SERF1* was induced under salinity in Empyrean 1 (Fig. 5). *HSP90.2* was induced in BB 106. *HSP90.7* was induced more than 2-fold in Empyrean 1 and Cornerstone. *SLAH1* was upregulated in Empyrean 1 and Bright’s Hybrid 5. *SLAH3* is upregulated in Empyrean 1, BB106, Bright’s Hybrid 5 (Fig. 5). It is worth mentioning that the expression levels of most genes were downregulated under salinity compared to the control in leaves of Guardian, one of the most salt-sensitive lines (Fig. 5). Lovell showed significant downregulation of *NHX2*, *AKT1*, *SLAH3*, *Prupe.6G205700* (Fig. 5).

It is noteworthy that some of the genes were downregulated in salt-tolerant lines under salinity and vice-versa. For example, genes including *HKT1*, *P5CS1*, *HSP90.2*, *OTS1*, *SGF29*, *ALMT9*, *NPF2.4*, *NPF2.5*, *SLAH1*, *SLAH3*, *CCC* were significantly downregulated under salinity in Cornerstone (Fig. 5). Additionally, *NPF2.4* and *NPF2.5* were significantly downregulated in Bright’s Hybrid 5. *CCC* was downregulated in Cornerstone, BB 106, and Bright’s Hybrid 5. *AKT1* was downregulated in Empyrean 1. Similarly, *SOS1*, *SOS2*, *AVP1*, *P5CS1*, *HSP90.7*, *OTS1*, *SGF29*, *ALMT9*, *NPF2.4*, and *SLAH1* were significantly upregulated in salt-sensitive line, Lovell (Fig. 5).

## Discussion

This study was undertaken to evaluate 14 almond rootstocks for their salinity tolerance and to understand the roles of physiological, biochemical, and genetic mechanisms regulating salt tolerance in *Prunus*. We have evaluated non-grafted rootstocks under mixed salt composition water treatments consisting of control (T1), Na-SO_4_ dominant irrigation water (T2), Na-Cl dominant irrigation water (T3), Na-Cl/SO_4_ dominant irrigation water (T4) and Ca/Mg-Cl/SO_4_ dominant irrigation water (T5). Irrigation waters dominant in Na (T2, T3, and T4) negatively impacted the survival rate and the trunk diameter of almond rootstocks in comparison to Ca/Mg-Cl/SO_4_ dominant irrigation water (T5) (Fig. 1a,b). Na-Cl dominant irrigation water (T3) caused most reduction in survival rate and trunk diameter, followed by irrigation waters dominant in Na-Cl/SO_4_ (T4) and Na-SO_4_ (T2), indicating that Na^+^ is the most critical ion during salinity stress. Our findings also revealed that the lower performance of plants under Na-Cl dominant irrigation water (T3) than under Na-SO_4_ dominant (T4) irrigation water could be due to an increased toxicity of chloride over sulfate in almond rootstocks (Fig. 1a,b). Hence, Na and, to a lesser extent, Cl concentration in irrigation water are the most critical ion toxicities for almond rootstocks. Our previous work showed that Na toxicity is known to play an important role during salt stress in salt-tolerant crops such as alfalfa^10,21^; however, Cl toxicity plays a major role in salt-sensitive crops like strawberries and avocado^22,23^. For some plants, such as faba beans, concentrations of both Na and Cl ions are critical during salt stress^24^.

Both survival rate data and trunk diameter data indicated that among the 14 almond rootstocks, Empyrean 1, Cornerstone, Bright’s Hybrid 5, BB 106, F x A had a high salt-stress tolerance whereas Lovell, Guardian, Krymsk 86, Rootpac 20, and Rootpac R had low ability to tolerate salinity stress (Fig. 1c,d). These observations are backed up by the fact that different rootstocks used for almonds come from different *Prunus* species and their hybrids. Our data suggested that peach hybrid, Empyrean 1 and peach-almond hybrids such as Cornerstone, Bright’s Hybrid 5, BB 106, F x A, Nickels, and Hansen 536, are more tolerant to salinity than peach based rootstocks (Lovell and Guardian), plum based rootstocks (Rootpac 20), peach-plum hybrids (Krymsk 86) and plum-almond hybrids (Rootpac R). Complex hybrids of peach-almond-plum-apricot (Atlas and Viking) are intermediate in salinity tolerance (Fig. 1c,d). Hence, the genetic background of a rootstock in terms of which *Prunus* species it came from is a critical factor in regulating its salinity tolerance.

Toxic levels of ions like sodium, chloride, and sulfate in saline soil, or experimental medium, disturb ion homeostasis in plants^25^. Salt-tolerant plants use different types of molecular machineries, which restrict the entry of toxic levels of ions into the plant systems, extrude to the soil, and store the toxic ions in the vacuole^8,9^. Salt-sensitive plants may lack efficient machineries that maintain ion homeostasis. Thus, the accumulation of ions in tissues is an indicator of the salinity tolerance ability of a plant^26^. Ion composition analyses indicated that, indeed, Empyrean 1 that showed the highest relative survival rate and relative change in trunk diameter also accumulated the least amount of Na in leaves (Fig. 1 and Fig. 2a). Similarly, Cornerstone, which showed the second-highest relative survival rate, also showed the low amount of Na accumulation (Fig. 1 and Fig. 2a). In contrast, Rootpac 20 showed the highest accumulation of Na in leaves and is among the top 3 for the highest mortality rate (Fig. 1c and Fig. 2a). Lovell was also among the top 3 highest accumulators of Na. These findings suggest that Na^+^ contributed to reduced growth and lethality under salinity stress. Also, Na^+^ accumulation data indicated that Empyrean 1, Cornerstone, Nickels, and Viking possess efficient molecular machineries that help to restrict Na^+^ accumulation. In contrast, the opposite may be true for the rootstocks that accumulated high Na^+^, such as Rootpac 20, Lovell, Rootpac R, and Hansen 536 (Fig. 2a).

Empyrean 1, one of the top performers for survival rate and trunk diameter, accumulated the least amount of Cl in leaves under salinity (Fig. 2b). As expected for poor performers under salinity, Rootpac 20 and Lovell accumulated large amounts of Cl in leaves under salinity. Although Lovell was among the top three Cl accumulators in Na-Cl dominant treatment (T3), it accumulated significantly less Cl in Na-Cl/SO_4_ (T4) and Ca/Mg-Cl/SO_4_ (T5) treatments (Fig. 2b) compared to other high-chloride accumulators. These findings suggest that SO_4_^2-^ may play a role in Cl^-^ homeostasis in Lovell, which requires further investigation.

Interestingly, for almond rootstocks, the five lowest accumulators for Na were also the lowest accumulators for Cl (Fig. 2), indicating that these rootstocks may have efficient machineries for Na^+^ and Cl^-^ homeostasis.

Potassium is an important macronutrient that plays many vital roles in plants, including pH homeostasis, osmotic balance, stomatal opening, and catalysis of many enzymatic reactions^27^. Hence, higher K concentration in tissues of a specific plant genotype during salinity stress is considered a desired trait. Salt stress negatively affects K content in plants by possibly inhibiting K^+^ uptake, and an increase of Na^+^ in the cytosol causes membrane depolarization that, in turn, induces efflux of K^+^ from the cytosol^28^. Tissue ion analysis indicated that all 14 rootstocks showed a reduction in tissue K content in response to saline treatment. As expected, top performers like Viking, Bright’s Hybrid 5, Cornerstone showed a lesser percentage reduction of total K^+^ concentration in leaves, whereas low performers like Lovell, Guardian, and Rootpac 20 showed a higher percentage reduction in tissue K (Fig. 2c). It is interesting to see that Rootpac 20, one of the low performers, showed the highest leaf K content among all rootstocks in all treatments (Fig. 2c). However, it shows a considerable reduction in leaf K concentration in all four salinity treatments (T1-T4) compared to the control (T1). Hence, our data suggest that unlike Na or Cl, where total tissue concentration under salinity was important, for K the percent reduction under salinity as compared to control is more critical.

Different rootstocks showed variation in tissue Ca concentrations in treatments, T2 to T4 but did not show much change in tissue Ca concentrations as compared to T1 in most genotypes (Supplementary Table S1). However, T5, which contained a high amount of Ca in irrigation water, displayed high tissue Ca as compared to T1 in most genotypes. In many plant species, the ability to maintain high Ca content in plant tissue under salinity is associated with salinity tolerance^10,29,30^. However, in almond rootstocks, we did not observe any association between tissue Ca concentration and salinity tolerance.

Photosynthetic parameters like photosynthetic rate (*Pn*), transpiration (*Et*), intracellular CO_2_ concentration (C*i*), and stomatal conductance (*gs*) are affected by salinity stress. Salinity stress-induced stomatal closure inhibits photosynthesis in glycophytes^31^. The general performance of a plant could be determined by gas exchange study during normal and stressed conditions. *Pn* had the highest correlation (R^2^ = 0.88) with trunk diameter, followed by *gs* (R^2^ = 0.63), then *SPAD* (R^2^ = 0.53) (Fig. 3). A high correlation between *Pn* and trunk diameter suggests that *Pn* is an important physiological parameter and can be used as an indicator of salinity stress in almond rootstocks (Fig. 3). Potassium plays key regulatory roles in stomatal regulation. Decreased K^+^ concentration in leaves, specifically in guard cells, could lead to stomatal closure that would inhibit transpiration^32^. The rootstocks that performed well for *Pn* in T3, including Bright’s Hybrid 5, Empyrean 1, Nickels (Supplementary Table S2) showed minor reductions of − 7.0%, 3.2%, and 6.6% for leaf K content in T3 as compared to T1, respectively (Supplementary Table S1). On the other hand, three inferior performers for *Pn* in T3, Rootpac 20, Guardian, and Lovell (Supplementary Table S2), displayed reductions of 37.0%, 30.0%, and 21.4% in leaf K concentration in T3 compared to T1, respectively (Supplementary Table S1). These observations demonstrate the importance of K^+^ homeostasis for the photosynthetic parameters during salinity stress.

It has been shown previously that antioxidant capacity (ORAC) and total phenolics are involved in salinity stress response in plants^10,33^. In almond rootstocks, ORAC and total phenolics changed in response to salinity without a clear trend across the rootstocks for their survival rates, change in trunk diameter, or their abilities to exclude Na or Cl (Supplementary Table S3). However, leaf proline concentrations, expressed as the ratio (T3/T1) of Na-Cl dominant treatment (T3) to the control (T1) in the 14 rootstocks, had a significant positive correlation with leaf Na and Cl concentrations and an inverse correlation to their survival rate (Fig. 4). These observations established that cultivars inherently low in proline and thus presenting a low T3/T1 ratio were more tolerant to salinity than cultivars presenting a high T3/T1 ratio. Thus, proline concentration could be used as a biochemical marker in *Prunus* to determine the ability of a genotype to exclude Na and Cl, which is directly related to plant growth under salinity. A previous report in a *Citrus* hybrid rootstock (*Citrus paradisi* Macfad. cv. Duncan *x Poncirus trifoliata* (L.) Raf.) evaluated proline effects when applied externally to plants submitted to drought^34^ and concluded that proline was involved in the expression of several genes encoding for antioxidant enzymes (APX, SOD, GR, and CAT). However, the authors only evaluated one cultivar, unable to discern the proline response of different citrus rootstocks as we did for almonds. Thus, if their cultivar is salinity sensitive, it makes sense that external proline would be used to mitigate drought effects and that proline also worked as a signal molecule to trigger genes associated with antioxidant enzymes in a supposedly sensitive citrus genotype. It would be interesting to evaluate the effect of external proline in gene expression of almond rootstocks when comparing a salt-sensitive with a salt-tolerant almond genotype as proline is assumed to be produced to mitigate salinity response, but its role in abiotic response is not yet elucidated^34,35^. Another interesting point is that studies correlating proline function with salt tolerance have mostly been done in growth chambers and not successfully verified under field conditions^35^. Nevertheless, proline accumulation in response to stress may not have adaptive significance but maybe a consequence of altered cellular metabolism triggered by salinity. Our results, obtained after 10 months of field experimentation, indicate that rootstocks with a low biosynthesis of proline, when submitted to salinity stress, performed better under salt stress and maybe better suited to tolerate salinity under field conditions than *Prunus* genotypes that responded to salinity with high proline biosynthesis.

Expression analysis was carried out for a set of 23 genes selected for their involvement in salt stress. These include genes known to be associated with Na^+^ efflux from root to soil (*SOS1*, and *SOS2*)^36^, genes involved in sequestration of Na^+^ in vacuoles (*NHX1*, *NHX2* and *AVP1*)^37^, genes important for retrieving Na^+^ from xylem (*AKT1* and *HKT1*)^38,39^, genes involved in antioxidants and organic solutes (*SERF1*and *P5CS1*)^40,41^, genes involved in signal transduction during salt stress (*HSP90.2*, *HSP90.7*, *OTS1*, *SAL1*, *SGF29, Prupe.6G205700, Prupe.3G273300* and *Prupe.1G586200*)^10,42–44^, genes involved in sequestration of Cl^-^ in vacuoles (*ALMT9*)^45^, genes involved in Cl^-^ exclusion (*NPF2.5*)^45^, genes involved in Cl^-^ efflux from roots to the xylem (*NPF2.4*, *SLAH1*, and *SLAH3*)^46–48^, and retrieval of Cl^-^ from root xylem (*CCC*)^45^.

The expression analysis revealed that some genes were induced in roots or/and leaves under salinity. However, the expression levels of most genes were high in salt-tolerant compared to salt-sensitive genotypes both under control (T1) and Na-Cl dominant salinity treatment (T3). For example, *SOS1*, *SOS2*, *AVP1*, and *SERF1* were expressed at a high level in roots of the four most salt-tolerant rootstocks (Empyrean 1, Cornerstone, BB 106 and Bright’s Hybrid 5) in T3 (Fig. 5). On the contrary, *HKT1* was expressed at a low level in roots of the four most salt-sensitive rootstocks (Lovell, Guardian, Rootpac 20, and Rootpac R) in T3 (Fig. 5). These observations suggest that although upregulation/downregulation of some genes under salinity is critical for salinity tolerance, the basic expression differences among different genotypes contribute more towards salinity tolerance in *Prunus* (Fig. 5). The possible explanation can be supported by the fact that an extensive interspecific diversity exists among different almond rootstocks, leading to basic expression differences among rootstocks.

Comparisons of expression levels of various genes in salt-tolerant genotypes display some striking differences among these genotypes, which suggests that although these genotypes are relatively tolerant to salinity stress, they vary in the component traits of the salt tolerance mechanisms (Fig. 5). For instance, *AVP1* and *AKT1* were expressed at a relatively higher rate in roots of Empyrean 1 in T3, whereas *HKT1*, *P5CS1*, *HSP90.7*, and *SGF29* and *NPF2.4* were expressed at a relatively higher rate in BB 106 (Fig. 5). Similarly, Lovell, a salt-sensitive genotype, had high expression of *SOS1*, *SOS2*, *AVP1*, *and OTS1* in leaves. Poor performance of Lovell under salinity may be attributed to low expression levels of *NHX2*, *HKT1*, *SERF1*, and *SAL1*. Salt tolerance of a specific genotype cannot be determined by a single trait, but it is the output of interactions among several component traits at the systems level, including genetic, biochemical, physiological, and morphological responses at a specific time point^9,49,50^.

This is the first comprehensive study focusing on morphological, physiological, biochemical, and genetic characterization of salinity stress in a large number of commercial almond rootstocks. Here, we identified salt-tolerant rootstocks that may help expand almond cultivation to new areas that are currently unsuitable due to salinity problems. The expression analyses of several candidate genes helped us in characterizing genotypes based on different components of the salt tolerance mechanisms. Also, our data strongly indicate that *Pn* and proline ratio can be used as markers to screen for salinity tolerance in *Prunus*. This information will be extremely valuable to almond breeders and geneticists in making crosses and combining different components of salt tolerance mechanism into a single genotype that may result in the development of a highly tolerant rootstock to salt.

## Materials and methods

### Experimental setup and salt treatments

The experiment was conducted at the United States Salinity Laboratory (USDA-ARS) in Riverside, CA. Non-grafted plants of 14 different rootstocks (Atlas, BB106, Bright’s 5, Cornerstone, Empyrean 1, Flordaguard x Alnem (F x A), Guardian, Hansen 536, Krymsk 86, Lovell, Nickels, Rootpac 20, Rootpac R, and Viking) were obtained from various nurseries and transplanted into 1.5-gallon pots containing 1:1 mix of sand:sandy loam soil. The experiment was set up in a randomized complete block design with 14 genotypes, 3 replications, 3 plants per replication (one plant per pot), and 5 treatments of saline water (total 630 trees). Plants were allocated into fifteen different blocks, each containing combinations of all genotypes and 3 replications. Blocks with different treatments were color-coded. The composition of five treatments is described in Table 1. These mixtures represent a range of natural saline water compositions, including municipal water as control. Water concentrations of the nutrients NPK were constant in all treatments. Treatment 1 was the control treatment with irrigation water Electrical Conductivity (E.C.) of 1.36 dS m^−1^ (municipal water + nutrients), and salinity of Treatments 2 through 5 were maintained at 3.0 dS m^−1^ despite their different ionic compositions. The pH of all irrigation water treatments was maintained between 7.3 and 7.6. Each plant was irrigated with 600 ml of treatment solution every other day.

### Trunk diameter and ion analysis

At the beginning of treatment, trunk diameter was recorded 10 cm above the soil level using a Vernier caliper. The second reading for the trunk diameter was recorded after ten months of treatment (control and salt treatments) to calculate the change. The survival rate of different rootstocks was also recorded after ten months of salt treatment. Leaf samples were collected 8 weeks after the initiation of salt treatments to determine tissue ion composition. Tissue samples were dried, digested in a Milestone Ethos E.Z. microwave digestion system, and analyzed with a Perkin Elmer Optima ICP OES for macro- and micronutrient elements. Chloride content was determined using the Labconco chloridometer. Statistical analysis was performed with the SAS software package.

### Physiological and biochemical analysis

Photosynthetic parameters and stomatal conductance were measured with a Li-Cor 6400 Photosynthesis System (Li-Cor Biosciences, Lincoln, NE, USA) 8 weeks from the initiation of the salt treatments. The measurement was conducted from 9:30 to 14:30, on sunny days from Aug. 17 to Aug. 24, 2017, under a condition of photosynthetic photon flux density 1400 μmol_photon_ m^-2^ s^−1^, operational or chamber ambient CO_2_ concentration 400 μmol_CO2_ mol_air_^−1^. Chamber temperature and leaf to air vapor pressure deficit ranged from 27.0 to 31.4 °C and from 1.57 to 3.41 kPa, respectively. Leaf chlorophyll content of each leaf used for the leaf gas exchange measurement was measured as Soil-Plant Analyses Development (SPAD) reading using a Chlorophyll Meter SPAD-502 (Minolta, Osaka, Japan). Fully expanded leaves in the top portion of the trees were chosen for the measurement. The replication was two leaves for one treatment replication for each rootstock, one leaf per tree; thus, the sample size was 6 leaves per salt treatment per rootstock.

For biochemical analysis, fully expanded leaves were collected from the top portion of trees. Leaves from three plants were combined as one sample for each treatment and each rootstock. The samples were immediately frozen in liquid nitrogen, then lyophilized in a Freeze Dry System (FreeZone 6, Labconco, Kansas City, MO) for 72 h. The dried samples were ground in a Wiley mill to pass a 20-mesh (0.635 mm) screen for analyses of proline, total phenolics, and hydrophilic antioxidant capacity [assayed as Oxygen Radical Absorbance Capacity (ORAC)].

Proline analysis was performed according to a previously validated procedure^51^, with some modifications. The ground leaf sample (250 mg) was added to 25 mL of deionized water for extracting proline. The samples were kept in a water bath at 45 °C for one hour, during which, tubes were mixed four times with a vortex at 15-min intervals. They were centrifuged at 5000 g for 15 min. The supernatant was filtered through 10 µm syringe filter (PP broad chemical compatibility type, Tisch Scientific, North Bend, Ohio), and 1 mL of the extract was taken and transferred to a glass test tube. To this tube, 1 mL of acid ninhydrin and 1 mL of glacial acetic acid were added separately and mixed vigorously at 375 RPM for 12 min using a platform shaker (Innova 2000, New Brunswick Scientific, Edison, New Jersey). The mixture was placed in a 100 °C water bath and boiled for 1 h. The reaction was then immediately stopped by placing the tubes in an ice bath. Toluene (2.0 ml) was added to the cooled tubes and mixed by stirring vigorously at 375 RPM for 12 min using the platform shaker. The upper phase (toluene) containing proline/ninhydrin chromophore compound was removed and transferred to a glass cuvette and was read at 520 nm in a spectrophotometer (DU 7500, Beckman Coulter, Brea California) using pure toluene as a blank. Leaf proline concentration was calculated from a standard curve on a dry weight basis.

The ORAC assay is based upon the inhibition of the peroxyl-radical-induced oxidation initiated by thermal decomposition of azo-compounds such as 2,2′-azobis (2-amidino-propane) dihydrochloride (AAPH)^52^. The ground freeze-dried leaf samples (0.5 g) were mixed with 5 g of sand. Each mixture was then extracted in a pressurized stainless steel cell (ASE 350, Dionex Corp.) using acetone:water:acetic acid (70:29.5:0.5 by volume) for the hydrophilic fraction. The sample extract was brought to 22.5 mL in the acetone–water-acetic acid solution, and a portion of it was taken and diluted for the analysis for their antioxidant capacity (ORAC) in triplicate in a 96 well plate using a FLUOstar OPTIMA (BMG LABTECH, Offenburg, Germany).

The same ASE 350 aqueous acetone extracts were used for quantification of total phenolics according to the Folin-Ciocalteu method^53,54^ using gallic acid (Sigma-Aldrich, Saint Louis, MO) as standard. The absorbance was read at 765 nm using a microplate spectrophotometer (xMark, BIO-RAD, Hercules, CA). Samples were analyzed in triplicates, and their total phenolics concentration was quantified against a gallic acid standard curve. Folin Ciocalteu phenol reagent stock solution was purchased from Sigma-Aldrich, Saint Louis, MO.

### Primer design for expression analyses

Almond genes involved in different mechanisms leading to salt tolerance were selected based on functional conservation with the genes identified in Arabidopsis^8,11,55^. These gene sequences were used in the Basic Local Alignment Search Tool (BLAST) analyses to identify corresponding sequences from the peach genome^19^. For each gene, the sequence with the highest homology was used, and intron/exon boundaries were identified. At least one PCR primer out of each pair was designed from two exons flanking an intron.

### Expression analyses

Tissue samples were taken 24 h after the initiation of salt treatment for RNA isolation. Young leaf and root samples were harvested from 630 plants (14 genotypes × 3 plants per genotype × 3 replications × 5 salt treatments). Samples from 3 plants for each genotype were pooled. Samples were frozen immediately in liquid nitrogen, and RNA was extracted using the Spectrum Plant Total RNA kit (Sigma, St. Louis, MO). To remove contaminating DNA, RNA was treated with DNase I following the manufacturer’s instructions (Thermo Scientific, Waltham, MA, USA). The qRT-PCR amplification was carried out in a BioRad CFX96 System using iTaq Universal SYBR Green One-Step Kit (Bio-Rad Laboratories, Hercules, CA, USA). Reactions for qRT-PCR were performed in 10 µl volume that contained 100 ng total RNA, 0.125 µl iScript Reverse Transcriptase, 0.75 µM of each of the primers, and 5 µl of 2 × one-step SYBR Green Reaction mix. The PCR program was as follows: 50 °C for 10 min, 95 °C for 1 min, then 40 cycles of 95 °C denaturation for 10 s, 57 °C annealing for 30 s, and 68 °C extension for 30 s^10^. For normalization of expression in different plates, four samples were used as inter-plate controls. The peach *EF2* and *Ubiquitin* genes were used as reference genes for the qRT-PCR analyses^56^. The cycle threshold values of each gene to the reference gene were used to calculate the relative expression, and differentially expressed genes were identified. For the quality control, the melt curve analysis was used to test the amplification specificity by ramping the temperature to 95 °C for 10 s, then back to 65 °C for 5 s, followed by incremental increases of 0.5 °C/cycle up to 95 °C.

## Supplementary information

Supplementary Information.

## Data Availability

All data supporting this study are included in the article and its supplementary material.

## Abbreviations

PCR: Polymerase Chain Reaction
qRT-PCR: quantitative Reverse Transcription-Polymerase Chain Reaction
ORAC: Oxygen Radical Absorbance Capacity
SPAD: Soil-Plant Analysis Development
BLAST: Basic Local Alignment Search Tool

## References

[1] NASS. National Agricultural Statistics Service. Non-citrus fruit and nut. https://downloads.usda.library.cornell.edu/usda-esmis/files/zs25x846c/0g3551329/qj72pt50f/ncit0520.pdf (2020).

[2] CDFA. California Agricultural Exports 2017–18. *California Agricultural Statistics Review, 2017–18*, 105–118. https://www.cdfa.ca.gov/statistics/PDFs/2017-18AgReport.pdf (2018).

[3] Fulton J, Norton M, Shilling F (2019). Water-indexed benefits and impacts of California almonds. Ecol. Indic..

[4] Howitt R, MacEwan D, Medellín-Azuara J, Lund J, Sumner DA (2015). Economic Analysis of the 2015 Drought for California Agriculture.

[5] Butcher K, Wick AF, DeSutter T, Chatterjee A, Harmon J (2016). Soil salinity: a threat to global food security. Agron. J..

[6] Wang W, Vinocur B, Altman A (2003). Plant responses to drought, salinity and extreme temperatures: towards genetic engineering for stress tolerance. Planta.

[7] Maas EV, Hoffman GJ (1977). Crop salt tolerance—current assessment. J. Irrig. Drain. Div. Am. Soc. Civ. Eng. ZDB.

[8] Munns R, Tester M (2008). Mechanisms of salinity tolerance. Annu. Rev. Plant Biol..

[9] Sandhu, D. & Kaundal, A. in *Biotechnologies of Crop Improvement, Volume 3: Genomic Approaches* (eds Gosal, S. S. & Wani, S. H.) 25–40 (Springer International Publishing, 2018).

[10] Sandhu D, Cornacchione MV, Ferreira JF, Suarez DL (2017). Variable salinity responses of 12 alfalfa genotypes and comparative expression analyses of salt-response genes. Sci. Rep..

[11] Gupta, B. & Huang, B. R. Mechanism of salinity tolerance in plants: physiological, biochemical, and molecular characterization. *Int. J. Genomics* **2014, Article ID 701596**, 18 pages. 10.1155/2014/701596 (2014). 10.1155/2014/701596 PMC3996477 24804192

[12] Sandhu D, Acharya B (2019). Mechanistic insight into the salt tolerance of almonds. Progressive Crop Consultant.

[13] Najafian S, Rahemi M, Tavallali V (2008). Effect of salinity on tolerance of two bitter almond rootstocks. Am.-Eurasian J. Agric. Environ. Sci..

[14] Gradziel TM (2020). Redomesticating almond to meet emerging food safety needs. Front. Plant Sci..

[15] Velasco D, Hough J, Aradhya M, Ross-Ibarra J (2016). Evolutionary genomics of peach and almond domestication. G3 Bethesda.

[16] Zrig A (2016). Effect of rootstock on sallinity tolerance of sweet almond (cv. Mazzetto). S. Afr. J. Bot..

[17] Zrig A (2015). A comparative study of salt tolerance of three almond rootstocks: contribution of organic and inorganic solutes to osmotic adjustment. J. Agric. Sci. Technol..

[18] Dejampour J, Aliasgarzadb N, Zeinalabedini M, Niya MR, Hervan EM (2012). Evaluation of salt tolerance in almond [*Prunus dulcis* (L) Batsch] rootstocks. Afr. J. Biotechnol..

[19] Verde I (2013). The high-quality draft genome of peach (*Prunus persica*) identifies unique patterns of genetic diversity, domestication and genome evolution. Nat. Genet..

[20] Ahmad R (2011). Whole genome sequencing of peach (*Prunus persica* L.) for SNP identification and selection. BMC Genomics.

[21] Cornacchione MV, Suarez DL (2017). Evaluation of alfalfa (*Medicago sativa* L.) populations’ response to salinity stress. Crop Sci..

[22] Sandhu D (2019). Variable salinity responses and comparative gene expression in woodland strawberry genotypes. Sci. Hortic..

[23] Suarez DL, Grieve CM (2013). Growth, yield, and ion relations of strawberry in response to irrigation with chloride-dominated waters. J. Plant Nutr..

[24] Tavakkoli E, Rengasamy P, McDonald GK (2010). High concentrations of Na^+^ and Cl^-^ ions in soil solution have simultaneous detrimental effects on growth of faba bean under salinity stress. J. Exp. Bot..

[25] Hanin M, Ebel C, Ngom M, Laplaze L, Masmoudi K (2016). New nnsights on plant salt tolerance mechanisms and their potential use for breeding. Front. Plant Sci..

[26] Negrão S, Schmockel SM, Tester M (2017). Evaluating physiological responses of plants to salinity stress. Ann. Bot..

[27] Ragel P, Raddatz N, Leidi EO, Quintero FJ, Pardo JM (2019). Regulation of K^+^ nutrition in plants. Front. Plant Sci..

[28] Almeida DM, Oliveira MM, Saibo NJM (2017). Regulation of Na^+^ and K^+^ homeostasis in plants: towards improved salt stress tolerance in crop plants. Genet. Mol. Biol..

[29] Hasegawa PM, Bressan RA, Zhu JK, Bohnert HJ (2000). Plant cellular and moleular responses to high salinity. Annu. Rev. Plant Physiol. Plant Mol. Biol..

[30] Ben Salah I (2009). Response of nitrogen fixation in relation to nodule carbohydrate metabolism in *Medicago ciliaris* lines subjected to salt stress. J. Plant Physiol..

[31] Bayuelo-Jiménez JS, Debouck DG, Lynch JP (2003). Growth, gas exchange, water relations, and ion composition of *Phaseolus* species grown under saline conditions. Field Crops Res..

[32] Hasanuzzaman M (2018). Potassium: A vital regulator of plant responses and tolerance to abiotic stresses. Agronomy.

[33] Boestfleisch C, Papenbrock J (2017). Changes in secondary metabolites in the halophytic putative crop species *Crithmum maritimum* L., *Triglochin maritima* L. and *Halimione portulacoides* (L.) Aellen as reaction to mild salinity. PLoS ONE.

[34] de Carvalho K, de Campos MKF, Domingues DS, Pereira LFP, Vieira LGE (2013). The accumulation of endogenous proline induces changes in gene expression of several antioxidant enzymes in leaves of transgenic *Swingle citrumelo*. Mol. Biol. Rep..

[35] Mansour MMF, Ali EF (2017). Evaluation of proline functions in saline conditions. Phytochemistry.

[36] Qiu Q-S, Guo Y, Dietrich MA, Schumaker KS, Zhu J-K (2002). Regulation of SOS1, a plasma membrane Na^+^/H^+^ exchanger in *Arabidopsis thaliana*, by SOS2 and SOS3. Proc. Natl. Acad. Sci. USA.

[37] Barragan V (2012). Ion exchangers NHX1 and NHX2 mediate active potassium uptake into vacuoles to regulate cell turgor and stomatal function in *Arabidopsis*. Plant Cell.

[38] Rubio F, Gassmann W, Schroeder JI (1995). Sodium-driven potassium uptake by the plant potassium transporter HKT1 and mutations conferring salt tolerance. Science.

[39] Peng Z (2016). Na^+^ compartmentalization related to salinity stress tolerance in upland cotton (*Gossypium hirsutum*) seedlings. Sci. Rep..

[40] Sun ZM (2016). Overexpression of the *Lotus corniculatus* soloist gene *LcAP2/ERF107* enhances tolerance to salt stress. Protein Pept. Lett..

[41] Nanjo T (1999). Antisense suppression of proline degradation improves tolerance to freezing and salinity in *Arabidopsis thaliana*. FEBS Lett..

[42] Ruibal C, Castro A, Carballo V, Szabados L, Vidal S (2013). Recovery from heat, salt and osmotic stress in Physcomitrella patens requires a functional small heat shock protein PpHsp16.4. BMC Plant Biol..

[43] Shi H, Liu W, Yao Y, Wei Y, Chan Z (2017). Alcohol dehydrogenase 1 (ADH1) confers both abiotic and biotic stress resistance in Arabidopsis. Plant Sci..

[44] Kim D-Y, Jin J-Y, Alejandro S, Martinoia E, Lee Y (2010). Overexpression of AtABCG36 improves drought and salt stress resistance in Arabidopsis. Physiol. Plant..

[45] Li B, Tester M, Gilliham M (2017). Chloride on the move. Trends Plant Sci..

[46] Li B (2016). Identification of a stelar-localized transport protein that facilitates root-to-shoot transfer of chloride in Arabidopsis. Plant Physiol..

[47] Qiu J, Henderson SW, Tester M, Roy SJ, Gilliham M (2016). SLAH1, a homologue of the slow type anion channel SLAC1, modulates shoot Cl ^−^ accumulation and salt tolerance in *Arabidopsis thaliana*. J. Exp. Bot..

[48] Li B (2016). AtNPF2.5 modulates chloride (Cl^−^) efflux from roots of *Arabidopsis thaliana*. Front. Plant Sci..

[49] Chen ZC (2017). A magnesium transporter OsMGT1 plays a critical role in salt tolerance in rice. Plant Physiol..

[50] Formentin E (2017). Salt tolerance in crops: not only a matter of gene regulation. Plant Physiol..

[51] Bates LS, Waldren RP, Teare ID (1973). Rapid determination of free proline for water-stress studies. Plant Soil.

[52] Prior RL (2003). Assays for hydrophilic and lipophilic antioxidant capacity (oxygen radical absorbance capacity (ORAC_FL_)) of plasma and other biological and food samples. J. Agric. Food Chem..

[53] Singleton VL, Rossi JA (1965). Colorimetry of total phenolics with phosphomolybdic-phosphotungstic acid reagents. Am. J. Enol. Vitic..

[54] Slinkard K, Singleton VL (1977). Total phenol analysis—automation and comparison with manual methods. Am. J. Enol. Vitic..

[55] Sandhu D (2018). Molecular characterization and expression analysis of the Na^+^/H^+^ exchanger gene family in *Medicago truncatula*. Funct. Integr. Genomics.

[56] Tong ZG, Gao ZH, Wang F, Zhou J, Zhang Z (2009). Selection of reliable reference genes for gene expression studies in peach using real-time PCR. BMC Mol. Biol..

