# Supplementary material for: Linking diverse salinity responses of 14 almond rootstocks with physiological, biochemical, and genetic determinants
Source: Sci Rep. 2020 Dec 3;10:21087. doi: 10.1038/s41598-020-78036-4 (PMC7712888; doi:10.1038/s41598-020-78036-4)
Supplement: Supplementary file 1 — Supplementary Information. [file 41598_2020_78036_MOESM1_ESM.pdf]

Linking diverse salinity responses of 14 almond rootstocks with physiological, biochemical, and genetic determinants

Devinder Sandhu, Amita Kaundal, Biswa R. Acharya, Thomas Forest, Manju Pudussery, Xuan Liu, Jorge F.S. Ferreira, Donald L. Suarez

Supplementary Fig. S1. Performance of 14 almond rootstocks under five ion combinations of irrigation waters. a. Relative survival rate. b. Relative change in trunk diameter. Error bars represent standard errors. Control, control treatment (E.C. = 1.4 dS m^{-1}); Na-SO_4, Na-SO_4 based irrigation water treatment (E.C. = 3.0 dS m^{-1}); Na-Cl, Na-Cl based irrigation water treatment (E.C. = 3.0 dS m^{-1}); Na-Cl/SO_4, Na-Cl/SO_4 based irrigation water treatment (E.C. = 3.0 dS m^{-1}); Ca/Mg-Cl/SO_4, Ca/Mg-Cl/SO_4 based irrigation water treatment (E.C. = 3.0 dS m^{-1})

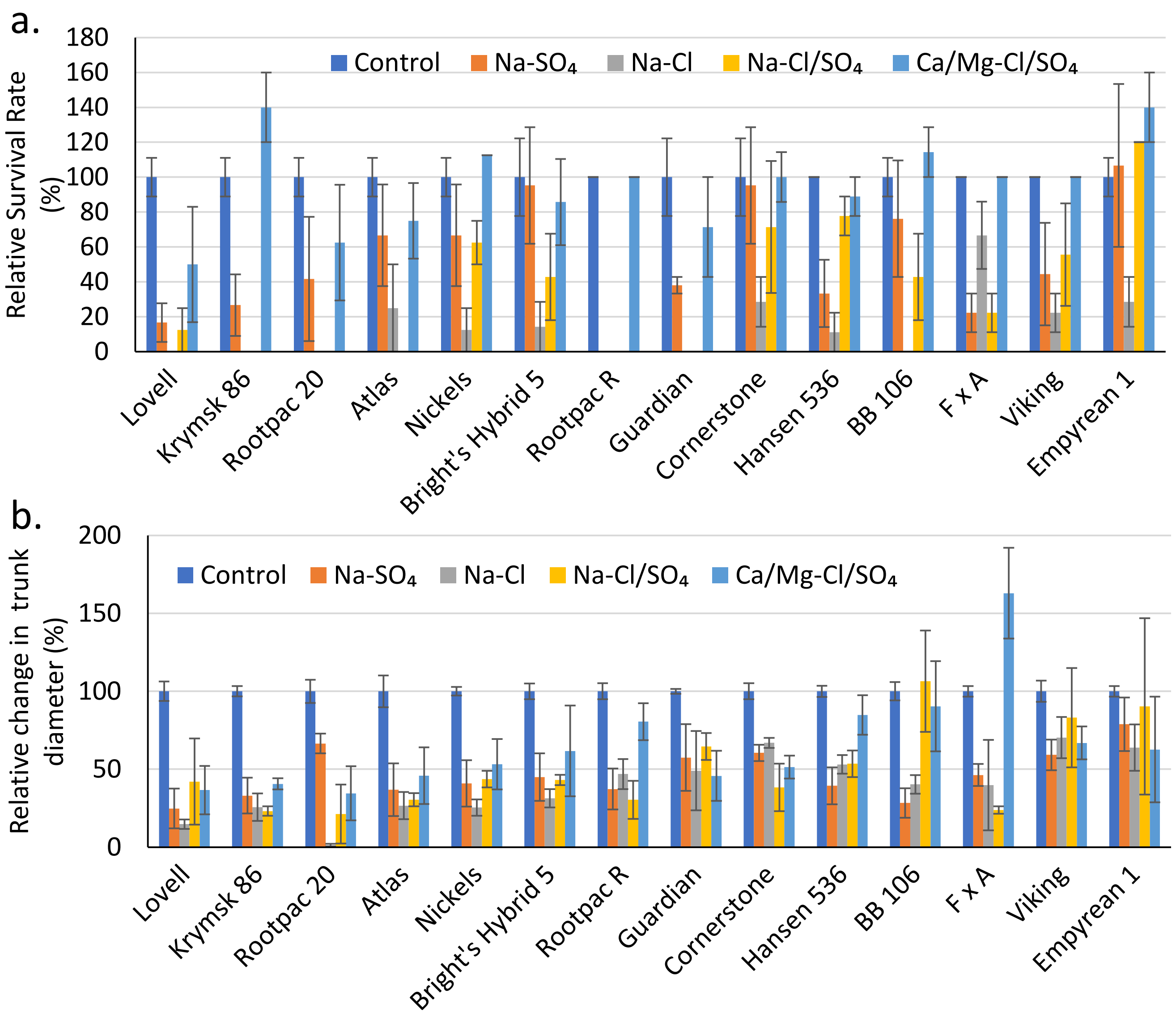

Supplementary Fig. S2. Leaf ion concentrations of 14 almond rootstocks treated with 5 irrigation water treatments. a. Na. b. Cl. c. K. Error bars represent standard errors. Means followed by the same letters are not significantly different according to LSD (0.05).

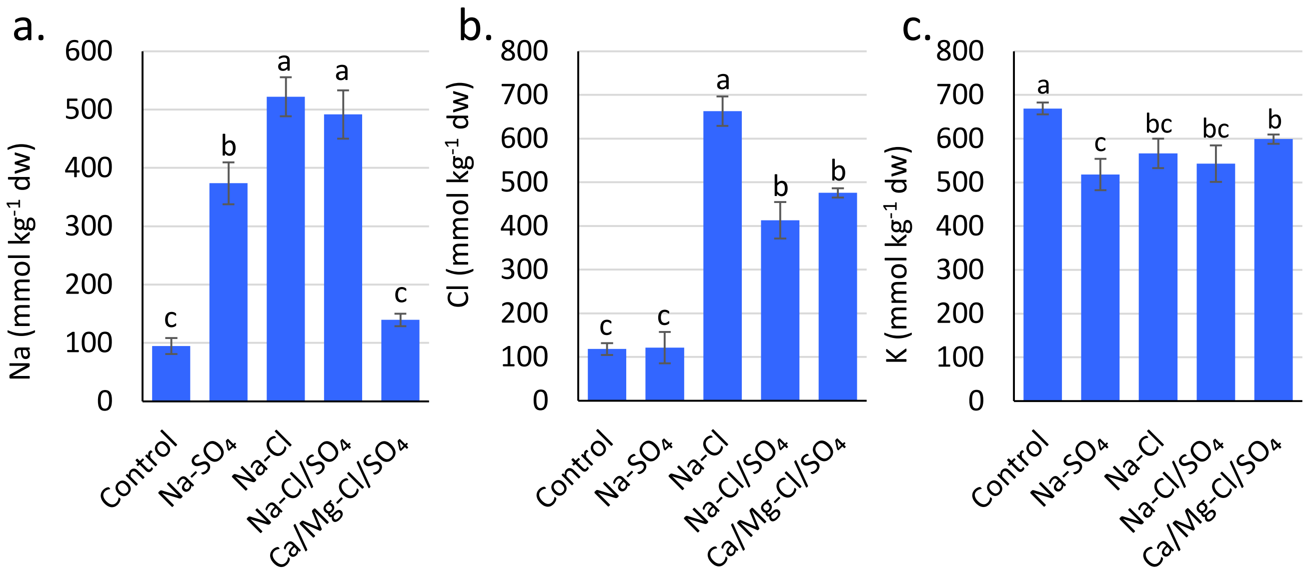

Supplementary Fig. S3. Biochemical parameters evaluated for 14 almond rootstocks treated with 5 irrigation water treatments. a. Oxygen Radical Absorbance Capacity (ORAC) . b. Total Phenolics. c. Proline.. Error bars represent standard errors. Means followed by the same letters are not significantly different according to LSD (0.05). TE, Trolox Equivalents; GAE, Gallic Acid Equivalents

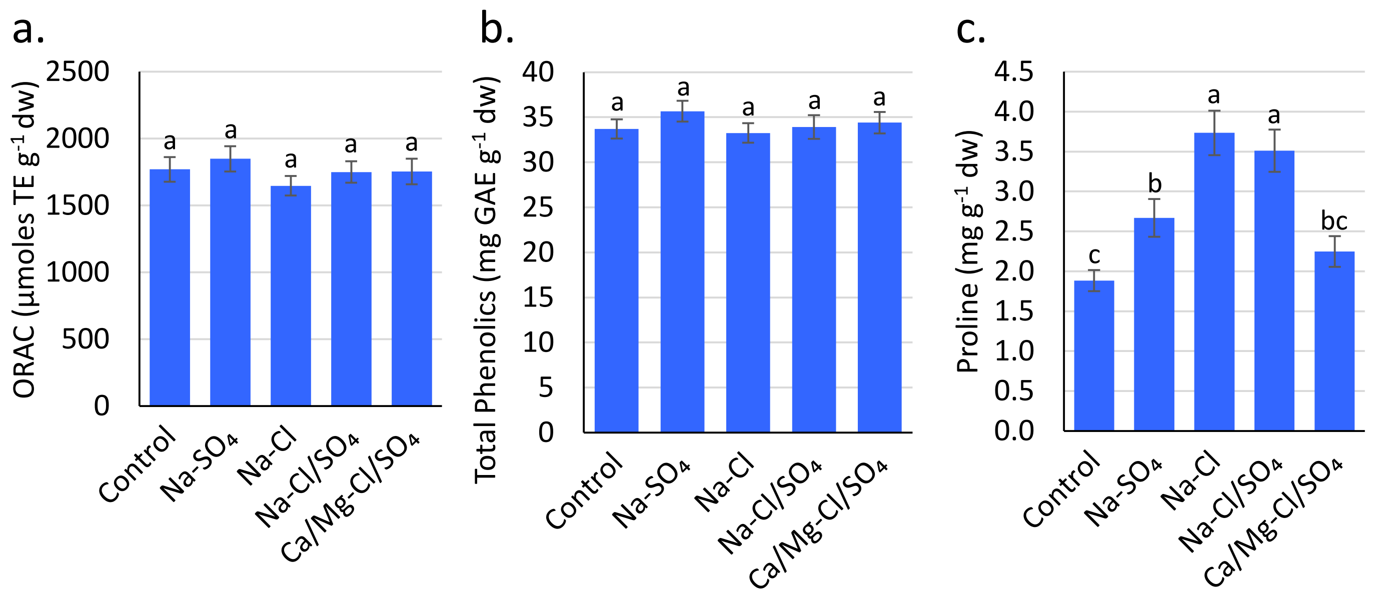

Supplementaty Table S1. Leaf ion cocentrations of almond rootstocks under five salinity treatments.

Tissue Na content (mmol kg^{-1} DW)

Genotype	Control (T1)		$Na\text{-}SO_4$ (T2)		Na-Cl (T3)		$Na\text{-}Cl/SO_4$ (T4)		$Ca/Mg\text{-}Cl/SO_4$ (T5)		Mean T2-T4	% increase
	Mean	SE	Mean	SE	Mean	SE	Mean	SE	Mean	SE		
Atlas	49.99	17.4	134.48	42.6	426.72	10.6	364.40	53.1	90.18	18.7	308.53	517.19
BB 106	131.16	54.2	317.27	40.7	378.03	63.4	393.37	51.2	199.45	41.6	362.89	176.68
Bright's Hybrid 5	159.62	89.3	194.94	66.2	503.61	71.0	404.85	97.6	121.96	33.8	367.80	130.42
Cornerstone	43.55	16.7	127.51	33.3	285.49	16.8	208.57	5.5	105.20	7.7	207.19	375.75
Empyrean 1	33.22	6.9	144.55	27.3	263.70	38.1	215.96	45.1	152.77	31.8	208.07	526.34
F x A	119.43	38.5	645.30	117.9	666.21	114.3	661.00	28.3	134.92	25.0	657.50	450.53
Guardian	104.97	11.3	415.05	12.9	632.50	41.4	457.56	83.7	188.67	65.2	501.70	377.95
Hansen 536	27.75	9.7	505.03	61.6	672.24	89.6	801.66	51.8	98.24	23.1	659.64	2277.09
Krymsk 86	206.27	69.7	495.20	48.0	612.69	32.9	598.00	67.0	174.80	29.8	568.63	175.67
Lovell	61.22	48.1	363.90	56.5	745.91	215.4	333.39	39.8	117.56	46.0	481.07	685.80
Nickels	68.76	56.3	281.23	88.4	286.72	76.4	272.57	36.8	224.38	48.3	280.17	307.47
Rootpac 20	136.95	85.6	863.52	45.6	791.40	60.2	997.52	176.2	83.08	46.6	884.15	545.60
Rootpac R	135.64	45.7	581.86	28.0	735.74	31.7	885.49	37.3	88.55	12.9	734.36	441.41
Viking	45.98	32.4	161.30	22.0	304.99	21.0	287.40	41.3	172.57	27.8	251.23	446.39

Tissue Cl content (mmol kg^{-1} DW)

Genotype	Control (T1)		$Na\text{-}SO_4$ (T2)		Na-Cl (T3)		$Na\text{-}Cl/SO_4$ (T4)		$Ca/Mg\text{-}Cl/SO_4$ (T5)		Mean T3-T5	% increase
	Mean	SE	Mean	SE	Mean	SE	Mean	SE	Mean	SE		
Atlas	101.66	15.0	82.48	19.7	551.41	11.6	325.99	33.9	423.59	33.6	433.66	326.58
BB 106	120.51	42.0	129.66	30.4	409.85	27.3	278.16	38.3	319.77	9.5	335.93	178.75
Bright's Hybrid 5	104.13	45.0	104.13	16.4	574.10	114.2	303.15	78.9	347.04	5.9	408.10	291.91
Cornerstone	88.70	12.8	80.61	4.3	439.87	31.6	204.81	11.8	407.12	211.7	350.60	295.26
Empyrean 1	54.98	8.0	50.05	7.0	359.21	64.5	175.86	8.7	243.00	32.8	259.36	371.73
F x A	97.06	19.2	137.93	12.2	626.02	25.7	348.91	22.3	339.67	44.0	438.20	351.47
Guardian	160.27	12.1	247.52	60.9	807.37	50.6	506.28	26.9	652.88	125.6	655.51	309.00
Hansen 536	156.85	23.8	153.68	7.9	1203.24	37.7	835.70	68.5	906.51	43.4	981.82	525.96
Krymsk 86	169.88	120.2	180.53	15.8	782.00	77.2	518.61	86.5	445.09	56.4	581.90	242.54
Lovell	46.19	32.8	93.04	22.1	872.64	45.7	297.60	40.6	341.74	30.3	503.99	991.13

Nickels	105.70	47.7	131.00	42.4	386.47	45.7	221.50	13.2	304.52	13.0	304.16	187.76
Rootpac 20	265.95	122.5	149.50	48.2	1146.78	112.0	888.04	143.3	928.82	110.7	987.88	271.45
Rootpac R	113.63	39.2	121.97	37.3	742.00	27.6	615.27	7.6	560.61	39.5	639.29	462.61
Viking	71.94	33.9	72.99	4.0	378.57	10.7	264.63	42.2	440.55	136.6	361.25	402.15

Tissue K content (mmol kg^{-1} DW)

Genotype	Control (T1)		$Na-SO_4$ (T2)		Na-Cl (T3)		$Na-Cl/SO_4$ (T4)		$Ca/Mg-Cl/SO_4$ (T5)		Mean T2-T5	% increase
	Mean	SE	Mean	SE	Mean	SE	Mean	SE	Mean	SE		
Atlas	621.32	46.2	456.16	43.9	524.43	29.4	547.05	25.7	576.02	50.8	525.92	-15.36
BB 106	549.39	37.2	411.83	60.1	521.22	31.3	446.08	82.8	479.91	37.8	464.76	-15.40
Bright's Hybrid 5	516.75	40.2	412.68	21.8	552.84	36.3	464.57	22.7	476.73	27.0	476.71	-7.75
Cornerstone	711.04	85.6	656.18	47.3	660.36	118.0	603.36	61.2	676.32	47.4	649.06	-8.72
Empyrean 1	429.30	60.7	373.89	21.8	415.79	16.0	351.48	34.3	366.74	7.8	376.98	-12.19
F x A	551.17	57.4	448.06	4.0	429.83	57.3	499.41	20.6	518.82	47.6	474.03	-14.00
Guardian	777.34	87.1	466.64	27.4	543.96	37.3	545.97	31.7	707.29	11.1	565.97	-27.19
Hansen 536	780.35	41.6	631.21	25.7	699.02	52.3	616.08	44.0	704.51	17.5	662.71	-15.08
Krymsk 86	704.82	12.8	510.70	36.6	637.99	53.6	655.29	19.1	713.05	49.8	629.26	-10.72
Lovell	601.98	30.9	412.00	42.8	473.43	83.3	422.59	76.8	507.98	61.7	454.00	-24.58
Nickels	531.78	8.0	475.12	14.2	496.57	53.2	393.62	31.8	482.62	29.7	461.98	-13.13
Rootpac 20	1295.71	49.0	910.85	44.8	816.32	38.3	911.46	23.0	1028.25	29.1	916.72	-29.25
Rootpac R	764.61	26.8	550.55	44.9	616.39	26.7	611.35	24.5	663.30	47.4	610.40	-20.17
Viking	529.18	56.9	538.15	12.4	535.14	19.6	534.51	42.7	483.34	42.6	522.79	-1.21

Tissue Ca Content (mmol kg^{-1} DW)

Genotype	Control (T1)		$Na-SO_4$ (T2)		Na-Cl (T3)		$Na-Cl/SO_4$ (T4)		$Ca/Mg-Cl/SO_4$ (T5)		Mean T2-T5	% increase
	Mean	SE	Mean	SE	Mean	SE	Mean	SE	Mean	SE		
Atlas	169.96	11.8	153.22	35.2	179.32	13.4	149.13	1.5	226.75	24.4	177.11	4.20
BB 106	287.64	45.2	255.54	27.6	262.03	20.0	269.55	20.9	330.02	23.6	279.29	-2.90
Bright's Hybrid 5	195.61	16.2	228.55	5.7	22.95	33.7	234.02	26.6	288.68	15.9	193.55	-1.05
Cornerstone	195.23	22.2	155.69	9.5	199.91	17.4	207.03	17.7	262.57	25.3	206.30	5.67
Empyrean 1	278.6	1.5	209.38	24.0	271.1	26.3	221.44	5.9	293.68	7.7	248.90	-10.66
F x A	328.81	17.4	328.49	9.2	358.62	13.7	344.47	18.5	404.67	19.5	359.06	9.20
Guardian	280.6	17.8	280.1	45.6	260.1	37.4	253.81	29.4	385.75	16.6	294.94	5.11

Hansen 536	286.69	16.7	247.93	13.3	287.56	21.6	298.57	20.5	455.08	43.0	322.29	12.42
Krymsk 86	269.25	73.4	260.57	17.0	268.5	27.1	294.02	16.1	389.73	31.0	303.21	12.61
Lovell	132.37	8.6	152.61	10.3	232.65	38.6	153.31	15.1	205.42	10.5	186.00	40.51
Nickels	338.46	37.8	265.04	20.9	363.8	10.7	337.47	6.4	360.57	13.4	331.72	-1.99
Rootpac 20	318.47	43.0	278.11	30.9	175.79	84.8	252.75	5.6	460.26	20.1	291.73	-8.40
Rootpac R	233.71	11.4	172.06	30.7	183.83	5.9	155.67	3.7	337.38	5.6	212.24	-9.19
Viking	201.41	32.9	227.02	25.6	208.21	23.7	232.78	12.2	409.36	123.7	269.34	33.73

Tissue Mg Content (mmol kg^{-1} DW)

Genotype	Control (T1)		Na-SO_4 (T2)		Na-Cl (T3)		Na-Cl/SO_4 (T4)		Ca/Mg-Cl/SO_4 (T5)		Mean T2-T5	% increase
	Mean	SE	Mean	SE	Mean	SE	Mean	SE	Mean	SE		
Atlas	138.11	5.7	130.63	17.2	142.12	9.3	122.75	7.3	214.93	17.3	152.61	10.50
BB 106	175.56	14.2	165.37	19.6	172.95	13.4	174.39	14.9	226.64	23.7	184.84	5.28
Bright's Hybrid 5	164.78	13.1	173.29	5.2	187.79	14.3	196.2	15.8	269.89	16.3	206.79	25.50
Cornerstone	148.24	11.2	143.86	11.9	174.01	13.9	161.65	5.1	195.05	14.7	168.64	13.76
Empyrean 1	190.34	4.4	154.25	10.7	174.5	13.4	148.32	4.8	221.7	8.7	174.69	-8.22
F x A	186.74	10.2	185.49	9.3	218.31	5.9	196.46	20.4	270.65	10.3	217.73	16.59
Guardian	206.1	10.7	201.95	10.9	185.23	17.8	194.42	20.8	278.47	14.2	215.02	4.33
Hansen 536	205.89	9.9	179.81	7.7	210.14	12.1	209.52	2.2	388.17	25.7	246.91	19.92
Krymsk 86	251.17	63.6	231.91	12.3	248.34	9.3	253.33	14.1	365.2	10.0	274.70	9.37
Lovell	128.15	1.8	136.85	4.9	185.76	12.6	135.95	7.7	191.66	9.7	162.56	26.85
Nickels	218.89	16.1	171.98	14.2	218.02	2.3	196.46	12.4	270.65	0.4	214.28	-2.11
Rootpac 20	229.41	19.8	186.29	21.3	178.93	8.4	178.3	11.0	421.17	19.9	241.17	5.13
Rootpac R	201.26	3.0	154.06	17.6	159.52	5.4	144.92	8.7	386.39	21.0	211.22	4.95
Viking	126.23	10.2	130.62	7.0	141.38	12.0	142.46	3.5	257.32	92.1	167.95	33.05

Tissue P Content (mmol kg^{-1} DW)

Genotype	Control (T1)		Na-SO_4 (T2)		Na-Cl (T3)		Na-Cl/SO_4 (T4)		Ca/Mg-Cl/SO_4 (T5)		Mean T2-T5	% increase
	Mean	SE	Mean	SE	Mean	SE	Mean	SE	Mean	SE		
Atlas	58.2	2.0	86.58	3.8	62.68	3.3	74.05	6.5	62.13	3.6	71.36	22.61
BB 106	26.22	3.1	25.55	2.1	27.92	1.7	28.15	1.5	27.52	1.7	27.29	4.06
Bright's Hybrid 5	25.34	1.7	31.6	2.2	33.33	3.8	30.79	3.5	29.14	2.3	31.22	23.18
Cornerstone	46.4	1.4	55.36	1.4	53.36	5.0	46.93	0.9	43.05	1.8	49.68	7.06

Empyrean 1	31.46	0.4	30.72	1.7	30.65	2.0	29.38	1.0	25.12	1.6	28.97	-7.92
F x A	44.77	7.9	38.6	1.4	37.41	3.0	46.32	7.0	40.53	1.6	40.72	-9.06
Guardian	56.77	3.6	54.02	9.4	80.18	12.3	65.57	10.7	61.17	10.3	65.24	14.91
Hansen 536	62.55	3.6	65.79	3.6	68.95	3.5	77.64	6.4	62.71	1.1	68.77	9.95
Krymsk 86	37.57	2.8	36.07	0.6	42.61	6.0	41.31	3.3	29.21	2.7	37.30	-0.72
Lovell	31.81	1.9	32.99	2.0	42.44	1.5	38.35	1.4	28.91	1.9	35.67	12.14
Nickels	35.46	1.9	30.93	1.1	34.05	4.7	34.6	4.9	25.68	1.5	31.32	-11.69
Rootpac 20	41.7	4.4	38.1	1.6	63.04	0.2	65.83	13.2	53.31	11.1	55.07	32.06
Rootpac R	41.65	3.6	57.06	6.0	56.73	8.3	70.74	4.6	47.79	3.3	58.08	39.45
Viking	28.79	0.8	30.09	2.0	26.84	2.0	31.92	1.0	39.6	9.2	32.11	11.54

Tissue S Content (mmol kg^{-1} DW)

Genotype	Control (T1)		$Na-SO_4$ (T2)		Na-Cl (T3)		$Na-Cl/SO_4$ (T4)		$Ca/Mg-Cl/SO_4$ (T5)		Mean T2-T5	% increase
	Mean	SE	Mean	SE	Mean	SE	Mean	SE	Mean	SE		
Atlas	59.84	2.1	91.43	4.4	46.72	0.7	66.77	1.7	81.65	4.9	71.64	19.72
BB 106	51.89	4.8	83.47	6.9	50.66	4.7	71.83	7.5	70.89	1.4	69.21	33.38
Bright's Hybrid 5	52.19	5.4	77.27	8.7	52.76	7.3	74.68	18.6	86.54	4.5	72.81	39.51
Cornerstone	62.33	3.5	89.91	4.3	66.27	4.6	74.23	3.6	76.11	2.1	76.63	22.94
Empyrean 1	42.72	2.0	66.92	5.9	46.25	5.3	52.68	2.3	68.57	12.6	58.61	37.18
F x A	52.21	4.4	122.94	16.2	48.68	3.6	62.71	3.1	67.54	4.3	75.47	44.55
Guardian	52.02	0.9	105.67	19.6	48.51	2.3	71.85	7.5	65.05	8.6	72.77	39.89
Hansen 536	94.49	7.5	301.49	18.4	59.89	4.0	156.85	8.2	157.33	12.1	168.89	78.74
Krymsk 86	77.51	14.5	113.91	7.9	55.27	2.2	85.44	2.4	80.94	7.6	83.89	8.23
Lovell	46.1	5.2	99.6	11.3	55.1	12.8	63.55	2.7	66.43	5.9	71.17	54.38
Nickels	52.05	4.3	94.67	18.2	55.3	5.1	62.33	5.2	73.42	2.7	71.43	37.23
Rootpac 20	129.7	26.8	357.97	19.4	64.66	10.7	175.88	30.9	162.24	31.7	190.19	46.64
Rootpac R	75.78	3.6	183.6	9.0	49.94	2.8	110.45	3.4	127.42	4.8	117.85	55.52
Viking	41.06	0.6	64.29	10.9	44.34	6.0	57.14	4.9	94.8	26.2	65.14	58.65

Tissue B Content (mmol kg^{-1} DW)

Genotype	Control (T1)		$Na-SO_4$ (T2)		Na-Cl (T3)		$Na-Cl/SO_4$ (T4)		$Ca/Mg-Cl/SO_4$ (T5)		Mean T2-T4	% increase
	Mean	SE	Mean	SE	Mean	SE	Mean	SE	Mean	SE		
Atlas	3.83	0.5	4.63	1.4	3.11	1.1	4.21	0.2	3.46	0.4	3.98	4.00

BB 106	3.48	0.2	2.68	0.4	2.47	0.8	2.78	0.3	3.01	0.4	2.64	-24.04
Bright's Hybrid 5	3.19	0.2	2.54	0.1	3.14	1.4	2.46	0.2	2.92	0.3	2.71	-14.97
Cornerstone	3.38	0.3	3.20	0.1	2.76	1.0	3.00	0.4	2.87	0.4	2.99	-11.60
Empyrean 1	4.37	0.3	3.55	0.2	2.81	1.1	3.31	0.5	3.26	0.2	3.22	-26.24
F x A	3.75	0.3	3.14	0.2	3.23	1.3	3.66	0.3	3.17	0.3	3.34	-10.84
Guardian	3.62	0.3	3.32	0.4	3.75	1.2	3.87	0.3	3.90	0.2	3.65	0.74
Hansen 536	4.66	0.1	3.43	0.2	2.73	1.1	3.61	0.3	3.91	0.1	3.26	-30.11
Krymsk 86	4.60	0.4	4.90	0.4	4.08	1.2	4.72	0.4	3.96	0.5	4.57	-0.72
Lovell	3.47	0.1	3.29	0.2	2.21	1.6	2.79	0.4	3.12	0.3	2.76	-20.37
Nickels	3.26	0.2	3.26	0.3	2.01	1.8	2.89	0.2	3.18	0.2	2.72	-16.56
Rootpac 20	3.99	0.1	3.59	0.4	4.81	1.0	4.40	0.6	4.93	0.2	4.27	6.93
Rootpac R	3.42	0.1	3.77	0.6	12.48	7.2	4.53	0.4	3.60	0.4	6.93	102.53
Viking	3.76	0.1	3.19	0.7	7.33	3.8	3.22	0.6	3.47	0.2	4.58	21.81

Tissue Cu Content (mmol kg^{-1} DW)

Genotype	Control (T1)		Na-SO$_4$ (T2)		Na-Cl (T3)		Na-Cl/SO$_4$ (T4)		Ca/Mg-Cl/SO$_4$ (T5)		Mean T2-T5	% increase
	Mean	SE	Mean	SE	Mean	SE	Mean	SE	Mean	SE		
Atlas	0.049	0.006	0.078	0.019	0.061	0.001	0.050	0.010	0.047	0.011	0.047	-4.26
BB 106	0.042	0.004	0.045	0.004	0.054	0.011	0.041	0.009	0.034	0.013	0.035	-17.10
Bright's Hybrid 5	0.035	0.006	0.048	0.007	0.046	0.012	0.037	0.009	0.031	0.009	0.032	-8.47
Cornerstone	0.040	0.003	0.040	0.006	0.048	0.009	0.035	0.012	0.026	0.004	0.030	-26.29
Empyrean 1	0.040	0.004	0.044	0.006	0.048	0.014	0.031	0.015	0.031	0.009	0.031	-23.18
F x A	0.072	0.014	0.071	0.008	0.073	0.016	0.049	0.012	0.057	0.008	0.050	-30.07
Guardian	0.043	0.006	0.046	0.005	0.047	0.013	0.041	0.013	0.026	0.011	0.032	-25.72
Hansen 536	0.046	0.002	0.054	0.006	0.065	0.011	0.054	0.009	0.040	0.013	0.043	-8.19
Krymsk 86	0.062	0.010	0.070	0.007	0.067	0.012	0.048	0.014	0.035	0.010	0.044	-29.03
Lovell	0.040	0.002	0.060	0.008	0.053	0.013	0.046	0.016	0.034	0.013	0.039	-4.46
Nickels	0.053	0.004	0.059	0.011	0.060	0.007	0.043	0.012	0.032	0.010	0.039	-26.98
Rootpac 20	0.076	0.005	0.107	0.004	0.110	0.007	0.089	0.019	0.068	0.006	0.075	-1.58
Rootpac R	0.074	0.007	0.102	0.008	0.094	0.015	0.073	0.020	0.061	0.002	0.066	-10.81
Viking	0.032	0.002	0.042	0.005	0.035	0.006	0.033	0.014	0.048	0.012	0.032	-1.44

Tissue Fe Content (mmol kg^{-1} DW)

Genotype	Control (T1)		$Na\text{-}SO_4$ (T2)		Na-Cl (T3)		$Na\text{-}Cl/SO_4$ (T4)		$Ca/Mg\text{-}Cl/SO_4$ (T5)		Mean T2-T5	% increase
	Mean	SE	Mean	SE	Mean	SE	Mean	SE	Mean	SE		
Atlas	4.59	1.1	4.67	1.2	3.26	0.8	3.49	0.1	3.73	0.1	3.79	-17.48
BB 106	4.71	1.0	3.78	0.3	4.18	0.4	4.32	0.4	3.86	0.2	4.04	-14.33
Bright's Hybrid 5	4.07	0.3	4.60	0.4	4.71	0.4	4.66	0.5	4.47	0.5	4.61	13.27
Cornerstone	4.58	0.7	4.29	0.5	4.85	0.7	5.00	0.3	4.94	0.2	4.77	4.15
Empyrean 1	3.99	0.3	4.04	0.4	4.04	0.1	3.99	0.6	3.92	0.5	4.00	0.19
F x A	5.01	0.1	5.32	0.4	4.99	0.6	4.94	0.2	4.86	0.3	5.03	0.35
Guardian	4.37	0.3	4.59	0.1	5.57	0.6	5.23	0.7	4.77	0.4	5.04	15.33
Hansen 536	3.57	0.3	4.17	0.4	4.21	0.5	5.04	0.5	4.02	0.3	4.36	22.13
Krymsk 86	3.98	0.3	4.25	0.2	4.63	0.8	5.36	0.5	4.53	1.2	4.69	17.90
Lovell	4.93	0.7	5.75	0.3	5.87	1.4	5.66	0.5	5.11	0.3	5.60	13.54
Nickels	4.03	0.7	3.96	0.8	4.55	0.5	3.53	0.6	3.76	0.2	3.95	-1.99
Rootpac 20	3.75	0.2	3.46	0.3	4.60	1.1	4.72	0.3	3.39	0.4	4.04	7.80
Rootpac R	4.27	0.0	4.83	0.4	4.56	0.8	3.77	0.4	5.11	0.3	4.57	6.97
Viking	3.56	0.3	3.93	0.0	4.20	0.6	4.88	0.3	4.40	0.3	4.35	22.26

Tissue Mn Content (mmol kg^{-1} DW)

Genotype	Control (T1)		$Na\text{-}SO_4$ (T2)		Na-Cl (T3)		$Na\text{-}Cl/SO_4$ (T4)		$Ca/Mg\text{-}Cl/SO_4$ (T5)		Mean T2-T4	% increase
	Mean	SE	Mean	SE	Mean	SE	Mean	SE	Mean	SE		
Atlas	0.49	0.0	2.20	2.2	0.49	0.0	1.19	1.0	1.22	1.0	1.27	162.63
BB 106	0.44	0.1	0.41	0.1	0.46	0.1	1.59	1.6	1.37	1.0	0.96	117.40
Bright's Hybrid 5	0.37	0.0	0.42	0.1	0.48	0.0	1.62	1.7	1.47	1.4	1.00	173.22
Cornerstone	0.45	0.1	0.49	0.1	0.58	0.0	1.63	1.5	2.75	1.5	1.36	203.45
Empyrean 1	0.62	0.0	0.52	0.1	0.50	0.0	1.52	1.5	1.39	1.1	0.98	57.38
F x A	0.84	0.1	0.77	0.1	0.97	0.0	1.92	1.5	2.02	1.5	1.42	69.34
Guardian	0.52	0.0	0.49	0.1	2.11	2.1	1.30	1.2	1.76	1.6	1.42	170.60
Hansen 536	1.96	0.7	1.67	1.0	0.90	0.1	2.15	1.6	1.70	0.6	1.60	-18.16
Krymsk 86	1.67	1.2	0.91	0.1	1.07	0.2	2.17	1.5	1.60	0.5	1.44	-13.92
Lovell	0.34	0.0	0.40	0.0	0.45	0.0	1.53	1.5	1.58	1.5	0.99	194.64
Nickels	0.51	0.0	1.05	0.8	0.60	0.1	1.48	1.4	1.38	1.1	1.13	119.55
Rootpac 20	1.38	1.2	0.65	0.0	1.86	1.7	2.63	1.4	1.43	1.1	1.64	18.93
Rootpac R	1.50	1.2	0.59	0.1	0.52	0.0	1.34	1.4	1.92	1.6	1.09	-27.18

Viking	0.46	0.1	0.50	0.1	1.54	1.4	1.50	1.3	1.47	0.9	1.25	169.77

Tissue Mo Content (mmol kg^{-1} DW)

Genotype	Control (T1)		Na-SO$_4$ (T2)		Na-Cl (T3)		Na-Cl/SO$_4$ (T4)		Ca/Mg-Cl/SO$_4$ (T5)		Mean T2-T5	% increase
	Mean	SE	Mean	SE	Mean	SE	Mean	SE	Mean	SE		
Atlas	0.009	0.001	0.018	0.013	0.009	0.002	0.011	0.005	0.010	0.007	0.012	33.33
BB 106	0.013	0.005	0.022	0.003	0.022	0.003	0.023	0.003	0.018	0.004	0.021	63.46
Bright's Hybrid 5	0.011	0.005	0.019	0.003	0.021	0.003	0.021	0.005	0.021	0.007	0.021	93.40
Cornerstone	0.007	0.001	0.010	0.002	0.012	0.003	0.013	0.008	0.003	0.001	0.010	35.71
Empyrean 1	0.029	0.003	0.039	0.004	0.035	0.005	0.030	0.007	0.033	0.006	0.034	18.10
F x A	0.015	0.003	0.027	0.003	0.028	0.005	0.018	0.008	0.014	0.004	0.022	45.00
Guardian	0.011	0.003	0.017	0.004	0.014	0.005	0.018	0.007	0.015	0.004	0.016	45.45
Hansen 536	0.001	0.002	0.007	0.006	0.011	0.002	0.010	0.007	0.010	0.000	0.010	955.56
Krymsk 86	0.041	0.003	0.055	0.011	0.045	0.007	0.044	0.008	0.038	0.004	0.046	10.98
Lovell	0.029	0.001	0.039	0.004	0.025	0.008	0.031	0.006	0.025	0.004	0.030	3.45
Nickels	0.011	0.002	0.024	0.006	0.023	0.004	0.015	0.004	0.014	0.004	0.019	73.41
Rootpac 20	0.029	0.001	0.050	0.004	0.031	0.004	0.032	0.006	0.032	0.002	0.036	25.09
Rootpac R	0.024	0.002	0.038	0.004	0.037	0.003	0.023	0.007	0.025	0.004	0.031	28.13
Viking	0.017	0.002	0.020	0.001	0.025	0.005	0.019	0.005	0.019	0.006	0.021	22.06

Tissue Content Zn (mmol kg^{-1} DW)

Genotype	Control (T1)		Na-SO$_4$ (T2)		Na-Cl (T3)		Na-Cl/SO$_4$ (T4)		Ca/Mg-Cl/SO$_4$ (T5)		Mean T2-T5	% increase
	Mean	SE	Mean	SE	Mean	SE	Mean	SE	Mean	SE		
Atlas	0.25	0.023	0.28	0.084	0.32	0.053	0.28	0.021	0.26	0.015	0.28	13.30
BB 106	0.17	0.017	0.16	0.003	0.21	0.015	0.16	0.012	0.16	0.004	0.17	1.32
Bright's Hybrid 5	0.15	0.005	0.15	0.019	0.23	0.035	0.15	0.019	0.16	0.005	0.17	15.00
Cornerstone	0.22	0.007	0.24	0.012	0.27	0.042	0.20	0.013	0.22	0.014	0.23	5.80
Empyrean 1	0.19	0.020	0.16	0.001	0.20	0.025	0.14	0.009	0.16	0.010	0.16	-14.08
F x A	0.19	0.035	0.21	0.037	0.17	0.018	0.16	0.025	0.21	0.031	0.19	-0.26
Guardian	0.22	0.010	0.24	0.028	0.29	0.018	0.29	0.031	0.20	0.005	0.25	15.34
Hansen 536	0.26	0.027	0.33	0.036	0.32	0.028	0.25	0.024	0.27	0.027	0.29	12.50
Krymsk 86	0.22	0.013	0.19	0.010	0.21	0.018	0.17	0.009	0.16	0.009	0.18	-17.27

Lovell	0.21	0.015	0.23	0.001	0.23	0.032	0.23	0.026	0.19	0.008	0.22	5.83
Nickels	0.17	0.010	0.17	0.015	0.19	0.008	0.21	0.024	0.14	0.019	0.18	3.82
Rootpac 20	0.24	0.007	0.29	0.020	0.36	0.004	0.30	0.054	0.28	0.022	0.30	26.56
Rootpac R	0.22	0.013	0.25	0.001	0.24	0.014	0.23	0.027	0.21	0.006	0.23	5.23
Viking	0.14	0.007	0.16	0.027	0.17	0.019	0.18	0.022	0.21	0.040	0.18	29.29

Supplementary Table S2. Physiologial parameters (Pn, gs, and SPAD) for 14 rootstocks under 5 different treatments of irrigation waters.

Pn (μmol CO_2 m^{-2} s^{-1})

Rootstock	Control		Na-SO_4		Na-Cl		Na-Cl/SO_4		Ca/Mg-Cl/SO_4	
	Mean	S.E.	Mean	S.E.	Mean	S.E.	Mean	S.E.	Mean	S.E.
Atlas	7.53	1.15	4.83	1.10	2.50	0.35	5.97	0.87	7.65	0.83
BB 106	8.15	1.14	3.94	1.68	3.75	0.64	5.33	0.87	5.98	2.16
Bright's Hybrid 5	9.78	0.54	3.61	1.40	5.07	1.28	3.97	0.84	4.72	0.86
Cornerstone	6.30	1.40	2.81	0.88	2.27	0.70	2.94	1.03	3.91	0.87
Empyrean 1	6.92	0.84	3.39	0.84	5.59	0.91	3.77	0.96	4.11	0.50
F x A	10.62	0.72	4.39	1.28	3.67	0.39	6.36	1.44	8.78	1.24
Guardian	4.73	1.08	5.51	1.30	2.77	0.64	4.35	0.96	2.57	1.08
Hansen 536	12.76	0.97	6.04	1.38	5.56	1.04	5.73	1.63	9.07	1.39
Krymsk 86	11.45	1.75	5.19	1.59	3.86	1.00	4.38	1.35	7.67	1.64
Lovell	6.33	1.76	1.39	0.38	2.21	0.63	2.73	1.13	3.90	1.32
Nickels	10.53	0.94	5.60	0.42	7.30	1.66	6.04	1.65	5.99	0.57
Rootpac 20	7.18	0.15	4.12	0.58	3.45	0.81	3.09	0.66	5.84	1.37
Rootpac R	10.08	0.93	2.98	0.55	3.76	0.35	1.85	0.64	7.06	1.10
Viking	4.75	0.75	3.66	1.01	3.77	0.51	4.30	0.62	6.42	0.93

gs (mol H_2O m^{-2} s^{-1})

Rootstock	Control		Na-SO_4		Na-Cl		Na-Cl/SO_4		Ca/Mg-Cl/SO_4	
	Mean	S.E.	Mean	S.E.	Mean	S.E.	Mean	S.E.	Mean	S.E.
Atlas	0.0908	0.0053	0.0588	0.0122	0.0412	0.0055	0.0918	0.0190	0.0693	0.0104
BB 106	0.1060	0.0198	0.0592	0.0267	0.0553	0.0085	0.0947	0.0177	0.0905	0.0287
Bright's Hybrid 5	0.1027	0.0142	0.0573	0.0187	0.0790	0.0212	0.0652	0.0264	0.0453	0.0093
Cornerstone	0.0743	0.0065	0.0612	0.0158	0.0443	0.0128	0.0587	0.0207	0.0442	0.0127
Empyrean 1	0.0983	0.0174	0.0412	0.0152	0.1003	0.0229	0.0533	0.0057	0.0535	0.0101
F x A	0.1618	0.0403	0.0847	0.0246	0.0707	0.0107	0.1958	0.0317	0.1374	0.0242
Guardian	0.0595	0.0154	0.0853	0.0173	0.0414	0.0106	0.0638	0.0073	0.0493	0.0169
Hansen 536	0.1378	0.0132	0.0852	0.0225	0.0900	0.0120	0.1067	0.0198	0.1227	0.0077
Krymsk 86	0.1495	0.0437	0.0982	0.0395	0.0835	0.0163	0.0987	0.0182	0.1350	0.0188
Lovell	0.0728	0.0104	0.0218	0.0044	0.0378	0.0105	0.0586	0.0174	0.0460	0.0096
Nickels	0.1895	0.0406	0.0707	0.0100	0.1302	0.0362	0.1080	0.0385	0.0938	0.0110

Rootpac 20	0.0955	0.0037	0.0563	0.0035	0.0608	0.0124	0.0550	0.0100	0.0930	0.0305
Rootpac R	0.1178	0.0176	0.0786	0.0245	0.0552	0.0059	0.0618	0.0120	0.0807	0.0145
Viking	0.1188	0.0324	0.0693	0.0210	0.0630	0.0061	0.0783	0.0250	0.0602	0.0058

SPAD reading

Rootstock	**Control**		**Na-SO_4**		**Na-Cl**		**Na-Cl/SO_4**		**Ca/Mg-Cl/SO_4**	
	**Mean**	**S.E.**	**Mean**	**S.E.**	**Mean**	**S.E.**	**Mean**	**S.E.**	**Mean**	**S.E.**
Atlas	39.30	4.37	41.25	4.52	38.17	2.63	42.28	1.87	48.33	2.71
BB 106	39.57	1.09	37.00	1.68	41.67	1.15	38.45	1.25	41.77	1.45
Bright's Hybrid 5	49.62	2.57	43.97	3.51	44.13	2.43	39.00	3.30	45.12	2.15
Cornerstone	43.75	1.66	43.70	2.90	43.18	2.53	42.17	2.57	38.82	1.02
Empyrean 1	43.08	1.96	33.92	3.02	38.62	1.78	37.00	1.36	41.27	3.09
F x A	42.27	1.72	38.28	2.76	35.83	2.60	36.58	4.30	42.78	1.47
Guardian	39.92	6.91	36.23	3.33	26.83	4.07	38.42	5.99	38.65	2.88
Hansen 536	51.27	2.16	47.42	2.48	52.07	3.40	52.85	3.86	49.03	2.23
Krymsk 86	38.18	2.30	40.65	3.11	35.52	3.78	40.62	2.39	44.77	1.59
Lovell	47.12	4.21	41.37	3.01	41.98	4.32	45.87	2.82	46.90	2.37
Nickels	38.85	2.21	35.80	1.11	42.28	1.86	38.92	3.11	40.77	2.08
Rootpac 20	36.60	1.01	37.40	3.20	25.42	3.34	27.75	4.24	31.83	1.25
Rootpac R	45.32	1.17	40.50	1.97	34.23	2.34	32.57	2.04	42.13	2.68
Viking	47.13	2.45	46.18	3.17	48.77	1.81	46.60	2.06	53.78	3.11

Supp Table S3. Oxygen radical absorbance capacity (ORAC) and total phenolics (PhenolH) evaluated in leaves of 14 almond rootstocks.

Rootstock	**ORAC** (µmoles TE g^{-1} DM)									
	Control		Na-SO_4		Na-Cl		Na-Cl/SO_4		Ca/Mg-Cl/SO_4	
	Mean	S.E.	Mean	S.E.	Mean	S.E.	Mean	S.E.	Mean	S.E.
Atlas	1201.30	204.76	1397.31	54.78	1433.35	23.05	1682.39	92.10	1512.12	62.58
BB 106	1715.79	48.28	1712.38	80.48	1473.64	70.59	1656.11	93.68	1690.98	109.25
Bright's Hybrid 5	1680.88	109.65	1676.66	62.59	1572.97	130.52	1566.26	163.88	1508.71	81.83
Cornerstone	1589.63	116.52	1694.53	113.36	1514.21	232.55	1619.54	119.04	1623.13	227.19
Empyrean 1	1358.69	59.90	1735.17	140.80	1809.68	296.23	1750.45	59.07	1963.96	60.15
F x A	1551.08	3.83	1387.36	135.29	1526.48	207.40	1660.09	260.40	1360.13	11.33
Guardian	1705.31	176.97	1656.61	78.35	1695.70	129.79	1841.82	190.24	1655.06	33.74
Hansen 536	1168.88	68.10	1001.02	103.38	939.28	125.49	913.45	154.57	838.74	76.88
Krymsk 86	2776.39	178.87	2932.71	331.37	2288.30	279.63	2429.02	184.34	2632.42	118.75
Lovell	2856.26	435.37	3069.52	97.65	2054.73	236.21	2584.69	66.63	2850.90	381.66
Nickels	1651.21	22.16	1748.05	117.61	1589.55	128.87	1492.79	139.55	1784.68	47.21
Rootpac 20	1823.34	143.62	1776.71	149.11	1619.37	244.93	1463.65	375.51	1600.16	67.35
Rootpac R	2527.89	214.49	2475.51	103.28	2215.73	380.46	2489.94	247.16	2370.43	478.27
Viking	1240.27	49.90	1471.80	88.67	1392.84	169.27	1347.12	61.03	1087.08	46.42

Rootstock	**phenolH** (mg GAE g^{-1} DM)									
	Control		Na-SO_4		Na-Cl		Na-Cl/SO_4		Ca/Mg-Cl/SO_4	
	Mean	S.E.	Mean	S.E.	Mean	S.E.	Mean	S.E.	Mean	S.E.
Atlas	35.63	2.61	38.91	3.61	42.50	1.43	44.77	2.99	34.50	1.84
BB 106	39.46	0.74	41.09	3.37	38.43	2.25	36.67	4.49	40.41	3.90
Bright's Hybrid 5	32.50	0.69	34.24	2.32	32.13	0.98	30.17	0.35	25.45	3.69
Cornerstone	31.56	1.34	34.22	0.84	31.39	3.06	31.77	1.42	38.31	2.76
Empyrean 1	30.13	1.17	32.82	0.24	34.20	2.37	33.57	2.03	37.68	1.41
F x A	30.27	1.73	33.41	1.30	31.51	2.83	33.73	4.98	32.47	0.06
Guardian	32.74	0.51	34.74	1.47	35.41	1.46	35.85	1.42	33.56	1.12
Hansen 536	22.77	0.92	21.24	3.17	19.72	0.77	19.45	0.62	21.06	1.73
Krymsk 86	44.40	4.30	47.72	2.41	40.55	0.89	38.82	2.83	44.00	1.58
Lovell	40.12	3.27	44.30	2.64	32.11	3.24	45.44	1.48	43.00	1.32
Nickels	37.19	1.12	37.63	2.83	36.04	3.15	33.03	5.96	37.53	0.64
Rootpac 20	34.97	2.74	35.38	3.28	31.85	5.23	28.27	7.46	31.82	3.43
Rootpac R	39.31	3.77	38.65	2.19	37.89	2.36	36.64	3.33	37.21	0.72
Viking	23.62	1.06	26.09	1.92	24.47	1.97	26.34	3.82	21.52	0.51

TE, Trolox Equivalents; GAE, Gallic Acid Equivalents

Supp Table S4. Survival rates, leaf Na and Cl concentrations and proline ratios of 14 almond rootstocks.

Rootstock	Survival rate (%)	Leaf Na (mmol kg^{-1})	Leaf Cl (mmol kg^{-1})	Proline content in T3 (mg g^{-1} dw)	Proline content in T1 (mg g^{-1} dw)	Proline Ratio (T3/T1)
Atlas	46.88	426.72	551.41	3.82	1.71	2.23
BB 106	64.29	378.03	409.85	3.19	3.08	1.04
Bright's Hybrid 5	64.29	503.61	574.10	2.37	1.29	1.84
Cornerstone	67.86	285.49	439.87	1.86	1.68	1.11
Empyrean 1	90.00	263.70	359.21	2.68	2.57	1.04
F x A	63.89	666.21	626.02	4.70	1.24	3.80
Guardian	25.00	632.50	807.37	5.71	2.54	2.25
Hansen 536	55.56	672.24	1203.24	6.00	2.13	2.82
Krymsk 86	40.00	612.69	782.00	4.34	0.97	4.49
Lovell	18.75	745.91	872.64	7.33	2.25	3.26
Nickels	56.25	286.72	386.47	1.48	1.41	1.05
Rootpac 20	25.00	791.40	1146.78	3.39	1.48	2.29
Rootpac R	30.56	735.74	742.20	3.72	1.38	2.70
Viking	58.33	304.99	378.57	3.24	2.07	1.56

Supplementary Table S5. Relative expression values of 23 genes in leaves and roots of 14 almond rootstocks under control (T1) and Na-Cl based treatment (T3).

Genes	Leaf																											
	Empyrean 1		Cornerstone		BB 106		Bright's Hybrid 5		F x A		Viking		Nickels		Hansen 536		Atlas		Krymsk 86		Rootpac R		Rootpac 20		Guardian		Lovell	
	T1	T3	T1	T3	T1	T3	T1	T3	T1	T3	T1	T3	T1	T3	T1	T3	T1	T3	T1	T3	T1	T3	T1	T3	T1	T3	T1	T3
SOS1	1.3	1.4	1.2	1.4	1.1	1.5	1.6	1.3	1.1	1.1	1.0	1.3	1.0	1.4	1.3	1.6	1.8	1.3	1.0	1.2	1.3	1.0	0.9	0.6	2.0	1.2	1.8	3.0
SOS2	1.0	1.4	1.2	1.2	1.1	1.4	1.2	1.2	1.3	1.2	1.7	1.4	1.0	1.1	0.7	0.8	1.1	1.2	1.1	1.0	1.0	1.0	1.4	1.3	3.0	1.7	1.7	2.9
NHX1	0.6	0.6	0.8	0.8	0.6	0.6	0.7	0.6	0.6	0.9	0.5	0.4	0.4	0.7	0.6	0.7	0.6	0.6	0.6	0.6	0.8	0.8	1.0	0.8	0.8	0.6	0.7	0.7
NHX2	1.7	1.7	0.7	0.9	1.2	1.2	0.9	0.8	0.7	0.8	0.8	1.1	1.3	1.3	1.3	1.0	1.5	0.8	1.2	1.1	1.8	1.9	2.2	1.7	1.0	0.7	1.0	0.5
AVP1	1.8	2.5	2.5	2.9	2.0	2.2	1.8	2.0	1.8	1.9	1.6	1.4	1.7	1.6	2.0	2.3	1.4	1.6	1.4	1.1	1.4	1.5	2.6	2.5	2.3	1.9	1.4	3.3
AKT1	0.8	0.4	0.5	0.4	0.5	0.4	0.6	0.5	0.9	0.6	1.1	1.0	0.7	1.0	0.4	0.2	0.2	0.2	0.3	0.1	0.1	0.0	0.2	0.0	0.6	0.4	0.4	0.4
HKT1	1.1	1.1	2.0	1.2	1.3	1.4	0.9	0.9	1.5	1.0	1.5	1.0	0.8	1.7	1.1	0.4	0.2	0.6	0.7	0.4	0.6	0.6	0.3	0.8	0.4	0.7	1.5	0.6
SERF1	1.9	2.5	2.5	2.3	2.1	2.3	2.3	2.0	2.4	2.7	2.4	2.3	1.8	1.8	1.9	2.2	1.6	1.8	1.8	1.7	1.8	2.0	5.8	5.3	2.9	2.0	1.6	1.6
P5CS1	1.3	1.1	2.5	1.4	0.9	1.0	1.7	1.6	1.7	1.9	1.2	0.8	1.7	0.8	1.0	1.2	0.8	1.0	2.1	1.1	1.1	1.0	3.1	2.6	2.1	0.9	0.6	1.3
HSP90.2	0.7	0.7	2.5	1.2	0.7	1.1	2.1	1.6	0.7	1.1	1.0	0.5	1.0	1.0	0.9	1.0	1.0	1.3	0.8	0.6	0.4	0.7	0.1	0.1	4.3	1.5	0.9	1.0
HSP90.7	0.4	0.8	0.3	0.7	0.6	0.6	0.7	0.5	0.7	1.1	1.6	2.3	0.6	0.7	0.4	0.9	1.3	0.6	1.9	1.3	0.9	1.1	2.2	2.3	3.9	1.7	0.4	2.0
OTS1	1.9	2.2	2.6	1.7	2.2	1.9	2.9	2.1	2.5	2.2	2.0	1.0	2.6	1.1	1.4	1.6	1.4	1.5	1.4	0.7	1.0	0.9	1.9	1.3	3.0	1.4	1.2	2.9
SAL1	1.5	1.7	1.8	1.5	1.7	1.6	1.6	1.2	1.1	1.1	1.7	1.2	1.1	1.0	1.2	1.3	1.4	1.3	1.7	1.2	1.5	1.2	2.2	1.5	1.7	1.9	1.1	1.0
SGF29	1.2	1.8	3.0	1.5	1.5	1.1	1.9	2.0	1.9	1.5	1.2	1.3	2.6	1.0	1.0	1.1	1.1	1.2	0.6	0.4	0.5	0.5	0.1	0.0	2.2	1.4	0.9	2.2
ALMT9	1.2	1.4	3.7	1.2	1.1	1.0	1.8	1.6	1.6	1.5	1.0	1.3	1.8	1.0	1.0	0.8	0.5	0.6	0.8	0.5	0.5	0.5	1.8	1.5	1.4	1.1	0.6	1.8
NPF2.4	0.8	0.7	1.6	0.5	0.6	0.6	0.8	0.4	0.9	0.9	0.5	0.3	0.5	0.3	0.5	0.4	0.3	0.2	1.0	0.5	0.3	0.4	5.5	4.1	0.6	0.3	1.4	2.2
NPF2.5	0.5	0.4	0.9	0.8	0.3	0.4	0.8	0.1	0.2	0.2	0.5	0.2	0.2	0.0	0.3	1.4	1.7	2.2	0.2	0.1	0.2	0.3	0.3	0.1	0.9	0.4	0.6	0.8
SLAH1	0.7	1.1	2.7	2.2	1.7	0.7	2.1	1.6	0.2	0.6	1.4	2.7	1.7	0.5	0.5	0.3	0.8	0.5	0.3	0.2	0.4	0.1	0.6	0.1	4.3	0.2	0.6	1.5
SLAH3	0.2	0.4	1.1	0.5	0.7	1.0	1.2	1.6	1.2	0.8	1.3	3.5	2.3	0.3	0.4	0.8	0.2	0.1	0.2	0.3	0.2	0.1	0.5	0.4	0.2	0.4	0.1	0.2
CCC	2.0	2.1	4.4	2.7	1.9	1.5	3.0	1.8	1.8	1.8	1.6	1.5	2.1	1.2	1.4	1.7	1.3	1.4	2.0	2.1	1.6	1.8	5.7	5.5	2.9	1.5	1.4	1.8
Prupe.6G205700	0.1	0.0	0.0	0.1	0.0	0.0	0.1	0.0	0.1	0.0	0.0	0.0	0.0	0.0	0.0	0.1	0.0	0.0	0.0	0.0	0.0	0.0	1.0	1.7	0.0	0.0	0.0	0.0
Prupe.3G273300	0.0	0.0	0.1	0.1	0.1	0.1	0.1	0.1	1.0	0.5	0.0	0.0	0.0	0.0	0.0	0.1	0.0	0.1	0.0	0.1	1.1	1.0	1.8	0.7	0.0	0.0	0.0	0.0
Prupe.1G586200	1.7	0.9	0.2	0.5	0.6	0.5	0.2	0.9	0.6	1.7	0.7	1.9	0.6	1.0	0.1	0.9	0.6	3.6	1.2	1.5	0.9	5.8	0.7	7.2	0.9	4.4	0.6	0.8

Genes	Root																											
	Empyrean 1		Cornerstone		BB 106		Bright's Hybrid 5		F x A		Viking		Nickels		Hansen 536		Atlas		Krymsk 86		Rootpac R		Rootpac 20		Guardian		Lovell	
	T1	T3	T1	T3	T1	T3	T1	T3	T1	T3	T1	T3	T1	T3	T1	T3	T1	T3	T1	T3	T1	T3	T1	T3	T1	T3	T1	T3
SOS1	1.7	2.0	1.1	2.2	1.6	2.0	2.2	1.7	1.6	1.6	1.3	1.6	1.3	1.3	0.9	1.6	0.6	1.2	1.3	2.3	0.5	1.0	1.0	1.1	1.2	0.5	1.1	1.3
SOS2	3.2	3.4	2.0	2.6	3.1	3.4	2.5	2.9	2.4	2.8	3.2	3.2	2.4	2.4	1.2	1.7	3.2	4.6	1.6	2.1	1.7	1.5	0.6	0.7	2.8	2.1	2.3	2.1
NHX1	1.1	1.3	1.6	1.9	1.1	1.4	1.2	1.9	1.2	1.7	1.0	1.1	1.1	1.3	1.8	1.4	1.5	1.6	0.9	1.1	1.2	1.2	4.8	5.8	0.9	1.8	0.9	0.9
NHX2	2.1	2.1	2.0	2.2	1.9	3.2	1.9	1.4	2.1	1.9	1.4	0.9	1.8	1.8	1.8	0.7	3.0	6.5	1.1	1.4	1.4	1.4	0.9	0.7	1.8	1.8	1.3	1.3
AVP1	1.5	2.6	1.9	2.9	1.6	1.9	3.2	2.1	2.4	2.4	1.4	1.6	2.0	2.1	0.9	2.1	1.4	0.8	1.4	2.5	1.3	1.5	2.1	1.9	1.7	0.6	1.8	1.8
AKT1	1.1	1.2	0.7	0.7	0.9	1.0	0.6	1.1	1.4	1.1	1.7	0.5	0.6	0.6	0.3	0.6	0.4	0.4	0.2	0.2	0.2	0.3	0.0	0.0	0.7	0.4	0.9	0.9
HKT1	0.5	0.4	0.0	0.3	0.4	0.9	0.9	0.6	0.6	0.7	0.4	0.2	0.1	1.1	0.2	0.2	0.8	0.1	0.9	0.4	0.1	0.1	0.1	0.2	0.0	0.2	0.6	0.1
SERF1	2.1	3.2	3.4	4.3	2.2	3.2	1.7	3.2	2.2	3.2	2.0	2.8	2.2	3.2	2.8	3.0	2.1	3.7	2.2	2.7	2.0	3.1	3.7	4.9	2.4	1.1	1.9	2.0
P5CS1	1.3	1.6	2.6	2.4	1.1	4.3	0.8	1.0	1.5	2.6	1.6	1.1	1.2	1.5	1.7	2.4	2.0	4.3	1.3	1.5	1.2	1.0	0.3	0.7	1.2	0.6	1.4	1.0
HSP90.2	0.6	1.7	1.0	2.0	1.7	3.6	2.3	2.5	1.1	2.0	1.0	1.6	0.7	1.9	1.4	2.1	1.1	3.7	0.4	1.7	1.9	2.5	0.1	0.2	4.0	1.4	0.9	0.9
HSP90.7	2.0	2.9	1.5	1.6	1.4	5.6	6.3	3.5	1.8	5.0	2.1	2.7	2.2	1.4	1.2	2.8	1.2	0.8	1.5	2.7	1.5	1.4	2.6	2.0	1.0	1.8	0.9	1.4
OTS1	3.3	2.2	2.7	2.4	2.0	3.3	5.7	2.3	2.2	3.1	2.8	2.3	2.7	2.0	0.7	1.2	1.2	2.2	1.0	1.3	0.6	0.7	0.3	0.2	1.7	1.1	1.3	1.1
SAL1	1.0	1.0	1.1	1.3	1.2	1.3	1.7	1.0	1.2	1.4	1.1	0.3	1.0	0.7	0.7	0.8	0.6	0.3	0.9	1.0	0.6	0.6	0.9	0.7	0.7	0.6	0.8	0.7
SGF29	1.7	2.9	3.1	3.7	1.7	6.9	1.7	3.1	1.3	3.0	1.9	3.0	2.1	2.5	1.1	3.3	1.2	7.3	0.6	1.0	0.7	0.7	0.0	0.0	2.2	2.7	0.5	1.9
ALMT9	2.7	1.7	2.1	2.1	1.6	4.9	3.7	2.4	1.4	1.3	1.5	1.3	1.5	1.9	0.8	1.8	1.1	1.9	3.6	2.0	1.2	1.2	0.6	1.3	2.4	1.2	1.4	1.5
NPF2.4	2.6	2.0	0.5	0.6	2.5	6.9	1.1	0.5	1.2	0.3	0.4	0.3	2.0	1.5	0.5	4.3	0.3	0.0	0.1	0.1	0.1	0.1	0.1	0.1	0.2	0.2	0.9	0.6
NPF2.5	0.0	0.0	0.0	0.0	0.0	0.0	0.0	0.0	0.0	0.0	0.0	0.0	0.0	0.0	0.0	0.0	0.0	0.0	0.0	0.0	10.4	0.0	0.0	0.0	0.0	0.0	0.0	0.0
SLAH1	0.8	0.6	0.8	0.1	0.3	0.6	2.1	0.8	0.4	0.4	0.6	0.5	1.9	0.6	0.1	0.1	0.0	2.0	1.5	2.7	0.1	0.4	0.1	0.1	0.2	1.3	0.5	0.3
SLAH3	0.3	0.4	0.5	0.4	0.4	2.8	1.8	4.6	0.6	0.4	0.5	0.8	2.0	0.6	0.1	0.8	0.0	0.1	1.5	0.9	0.0	0.7	0.0	0.2	0.2	0.3	0.1	0.3
CCC	2.7	2.8	4.9	4.0	2.1	4.5	3.8	3.2	1.8	3.5	2.4	2.1	2.3	3.2	2.0	0.7	1.6	12.9	2.3	2.7	4.0	3.4	2.8	4.4	2.8	4.0	1.5	1.8
Prupe.6G205700	1.5	0.8	3.7	3.0	2.8	3.9	32.4	0.1	0.6	0.9	2.2	0.8	3.9	1.8	4.3	3.1	1.6	0.0	0.9	1.0	0.0	0.5	3.4	6.6	1.0	0.6	1.4	0.4
Prupe.3G273300	0.0	0.0	0.6	0.0	4.6	1.1	7.2	0.9	0.0	0.7	0.0	0.0	0.2	1.2	0.5	0.0	0.0	0.1	0.3	0.0	0.0	0.9	0.1	0.0	0.0	0.0	0.0	0.0
Prupe.1G586200	1.5	2.5	1.8	2.9	1.7	1.7	3.3	1.7	1.4	4.1	0.7	1.7	2.2	1.5	2.1	0.8	1.1	0.6	0.9	1.7	4.4	1.3	6.0	7.2	0.7	1.5	0.8	1.7
